# Supplementary material for: Efficient overall water splitting in acid with anisotropic metal nanosheets
Source: Nat Commun. 2021 Feb 16;12:1145. doi: 10.1038/s41467-021-20956-4 (PMC7887272; doi:10.1038/s41467-021-20956-4)
Supplement: Supplementary file 1 — Supplementary Information [file 41467_2021_20956_MOESM1_ESM.pdf]

Efficient overall water splitting in acid with anisotropic metal nanosheets

Dongshuang Wu[1*], Kohei Kusada[1*], Satoru Yoshioka[2], Tomokazu Yamamoto[2], Takaaki Toriyama[3], Syo Matsumura[2,3], Yanna Chen[4], Okkyun Seo[4], Jaemyung Kim[4], Chulho Song[4], Satoshi Hiroi[4], Osami Sakata[4], Toshiaki Ina[5], Shogo Kawaguchi[5], Yoshiki Kubota[6], Hirokazu Kobayashi[1], Hiroshi Kitagawa[1*]

1Division of Chemistry, Graduate School of Science, Kyoto University, Kitashirakawa-Oiwakecho, Sakyo-ku, Kyoto 606-8502, Japan.

[2]Department of Applied Quantum Physics and Nuclear Engineering, Kyushu University, Motooka 744, Nishi-ku, Fukuoka 819-0395, Japan.

[3]The Ultramicroscopy Research Center, Kyushu University, Motooka 744, Nishi-ku, Fukuoka 819-0395, Japan.

[4]Synchrotron X-ray Group and Synchrotron X-ray Station at SPring-8, National Institute for Materials Science, Kouto, Sayo-cho, Sayo-gun, Hyogo 679-5148, Japan

[5]Research & Utilization Division, Japan Synchrotron Radiation Research Institute (JASRI), SPring-8, Kouto, Sayo-cho, Sayo-gun, Hyogo 679-5198, Japan.

[6]Department of Physical Science, Graduate School of Science, Osaka Prefecture University, Sakai, Osaka 599-8531, Japan.

Experimental procedures

Chemicals. Poly(N-vinyl-2-pyrrolidone) (PVP, K30, MW ≈ 40000), BN powder, $RuCl_3 \cdot nH_2O$, $IrCl_4$, triethylene glycol (TEG), 95–98% concentrated H_2SO_4, 70% $HClO_4$, hydrogen chloride, diethylene ether, ethanol, and isopropanol were purchased from Wako (Japan). Nafion (5 wt.%) was bought from Sigma-Aldrich (USA). Conductive IrO_2 was purchased from Premetck Co. Ltd. (USA). All the chemicals and reagents were used as received without further purification. Doubly distilled deionized water (18.2 MΩ) was used for all synthetic preparations.

Synthesis of RuIr-NS. RuIr-NS with an average size of approximately 3.9 nm were synthesized by dissolving $RuCl_3 \cdot nH_2O$ and H_2IrCl_6 in a PVP-containing TEG solution at RT. Then, the mixture solution was heated from RT. to 230 °C and maintained at 230 °C for 3 h. The amounts of chemicals and agents used for synthesizing **RuIr-NS** were kept the same as those used for synthesizing **RuIr-NC**.

RuIr NPs with a large crystal size were obtained by a two-step process. First, RuIr NPs were obtained by the same process as that used for **RuIr-NS** except that PVP was not added. After obtaining the NPs powder, we heated the powder at 500°C for 5 min under vacuum to increase the crystal size. This sample is denoted as **RuIr-L**. Pure Ru NPs and Ir NPs were prepared by the same process as that of **RuIr-NS** using $RuCl_3 \cdot nH_2O$ and H_2IrCl_6, respectively.

HAADF-STEM 3D tomography. The tilt series of HAADF-STEM images for the individual **RuIr-NC** was acquired at the angle range from −65° to 65° with an angle step of 5° using the JEM-ARM200F instrument at 120 kV. The large tilt angle step was chosen in this study to avoid nanoparticle shape changes induced by electron irradiation. The acquired image size was 1024 × 1024 pixels at a pixel size of 0.1 × 0.1 $Å^2$. Eight images were acquired at each angle step with a short pixel time of 5 µs px^{-1} to minimize image distortion because of specimen drift. The image series at each angle step were averaged, the image size was reduced to 512 × 512 pixels, and the images underwent image alignment and affine transforms to correct image distortion because of specimen drifts. The background of each projection was removed after alignment using the cross-correlation method. Tomographic reconstruction was performed by the discrete

algebraic reconstruction technique (DART)[1, 2] in the ASTRA toolbox 1.8 of the MATLAB software developed by W. V. Aarle et al.[3] DART is an iterative algorithm that combines a continuous iterative reconstruction and discretization process for discrete tomography. DART is also capable of accurate reconstructions from a small number of projection images if a target 3D object has discrete intensity [2]. The reconstructed data were visualized via Visualizer Kai postprocessing software (Systems in Frontiers Inc.).

Quantitative analysis of the Ru/Ir ratio in the atomic-resolution HAADF-STEM images. To determine the Ir positions and thickness in the atomic-resolution HAADF-STEM images of **RuIr-NC**, atomic column intensities were quantitatively analysed with the Gaussian shape peak approximation using the StatSTEM library in MATLAB software developed by Backer et al.[4] The single Ru atom intensity was carefully estimated using single Ru atoms on a carbon film and matching image simulation based on the multislice method[5]. The single Ir atom intensity was also estimated as the Ru/Ir intensity ratio using the multislice image simulation. Ir positions were determined as intensity jumps compared with surrounding atomic column intensities.

X-ray absorption near-edge spectroscopy (XANES) analysis. XANES analysis was performed using the Athena program (version Demeter 0.9.26) in the IFEFFIT software package[6]. The pre-edge and post-edge background removal used a simple line and three-term polynomial regression subtraction, respectively. Approximately 50–80 eV above the edge, we took an inflection point in oscillation as the normalization point, the step height was made equal to 1, and the resulting curve was then normalized by dividing the jump of the absorption at the edge, which is the standard process for both the reference and the probed samples.

Principal component analysis (PCA). In this work, PCA and target transformation (TT) were used to determine the number and type of principal components in the sets of 13 XANES spectra (spectra at the OCP and from 1.25 V to 1.80 V, Supplementary Fig. 25)[7]. The serials of XANES spectra changing with potential in both RuIr samples showed two isosbestic points at ca. 22135 and 22157 eV suggesting that these spectra were generated from different mixtures of only two components. Using PCA, the number of probable species contained in these spectra was

also determined to be two. By using two components, we can reproduce the experimental data with a residual of less than 1% (two examples are shown in Supplementary Fig. 26). TT was adopted to identify the two possible components from the sets of six reference compounds, namely, Ru bulk, RuO_2, RuO_4 (water solution), ruthenium(III) acetylacetonate, $Ru_3(CO)_{12}$, and $RuCl_3$. Two model compounds were found to yield a sufficient match upon transformation, namely, Ru bulk and RuO_2 (Supplementary Fig. 27a,b). TT is powerful in identifying species that were not contained in the samples. As shown in Supplementary Fig. 27c–f, TT excludes the possibility of these references. Moreover, according to the Roubaix diagram of ruthenium[8], RuO_2 and its hydroxide forms must exist after being exposed to a voltage above 1.2 V in an H_2SO_4 electrolyte with a pH of 1. Therefore, according to both the statistics and physical meaning in the 13 XANES measurements of RuIr catalysts, Ru and RuO_2 are the two components.

Linear combination fitting (LCF). PCA and TT suggest that the sets of XANES spectra can be analysed by LCF using Ru and RuO_2. After careful spectral calibration and alignment (E_0 was chosen as the first peak of the first-derivative normalized XANES spectrum). The fitting was performed across the relative energy range of from −20 to 60 eV in the normalized XANES spectrum[7]. The spectral weight was forced to be between 0 and 1. All the weights sums were forced to equal 1. The E_0 values of all the standard references are fixed during the fit. The goodness-of-fit was judged by the residual factor (R-factor) and χ^2 values. Supplementary Fig. 28 gives examples of simulated and experimental results for the RuIr catalysts at 1.25 V, 1.40 V, and 1.80 V. All the fitting results are presented in Supplementary Table 3.

Extended X-ray absorption near-edge spectroscopy (EXAFS) analysis of the Ir *L*3-edge. EXAFS analysis was performed using the Artemis program (version Demeter 0.9.26) in the IFEFFIT software package[6]. The fitted k range was between 3 and 16 for all catalysts. The amplitude reduction factor (S_0^2) was 0.75(7) and was obtained by fitting the Ir bulk powder pellet with a fixed coordination number, which was applied for all the Ir L_3-edge analyses.

Electrochemically active surface area (EASA) evaluation on as-prepared particles. For the Cu UPD experiments[46], first, high-purity Ar was bubbled through the H_2SO_4

electrolyte (0.5 M) for at least 15 min to remove the O_2 in the electrolyte. After Ar saturation, the RuIr catalysts underwent electrochemical pre-treatment by potential cycling between 0.05 and 0.95 V for 200 cycles at a scan rate of 500 mV s^{-1}. Then, two CVs cycling between 0.05 and 1.00 V were recorded at 10 mV/s and used as the blank reference for Cu UPD experiments. Next, the working electrode was maintained at a certain deposition potential in an H_2SO_4 electrolyte (0.5 M) containing CuSO4 (5 mM) for 100 s. The deposition potential was determined as the potential just above the bulk Cu deposition. Afterwards, an LSV was measured from the deposition potential to 1.00 V with a scan rate of 10 mV s^{-1}. EASA values were evaluated from the Cu desorption charge of the linear background-corrected desorption performance. The measured charge was normalized by using the theoretical value of 0.42 mC cm^{-2} for a two-electron transfer assuming the oxidation of one Cu molecule to Cu^{2+} per metal atom.

To verify the accuracy of Cu UPD method, we also evaluate the surface area of **RuIr-NS** by the TEM based on a spherical geometry[9].

$$A_s \approx \frac{\sum \pi d^2}{\sum (1/6)\,\rho \pi d^3} = \frac{6 \sum d^2}{\rho \sum d^3} = \frac{6}{d_{v/a}/\rho}$$

where ρ is the bulk density of **RuIr-NS**, d is the average size determined by TEM, dv/a s the volume/area average diameter. We accounted for at least 200 NPs for evaluating A_s.

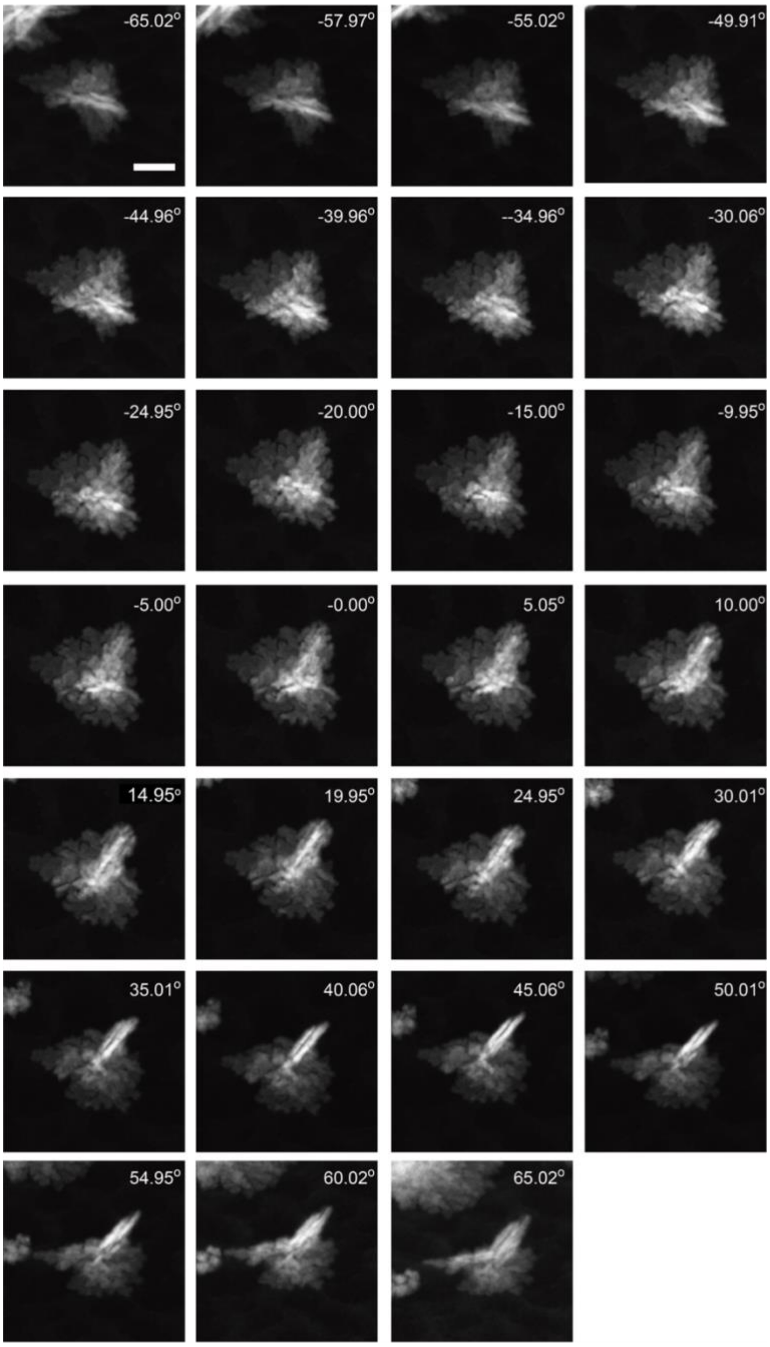

Fig. 1. Experimental tilt series obtained from one RuIr-NC particle. There were 27 projection images with a tilt range from −65.02˚ to +65.02˚ measured using a JEM-ARM200F instrument operated at 120 kV. The scale bar is 20 nm for all images.

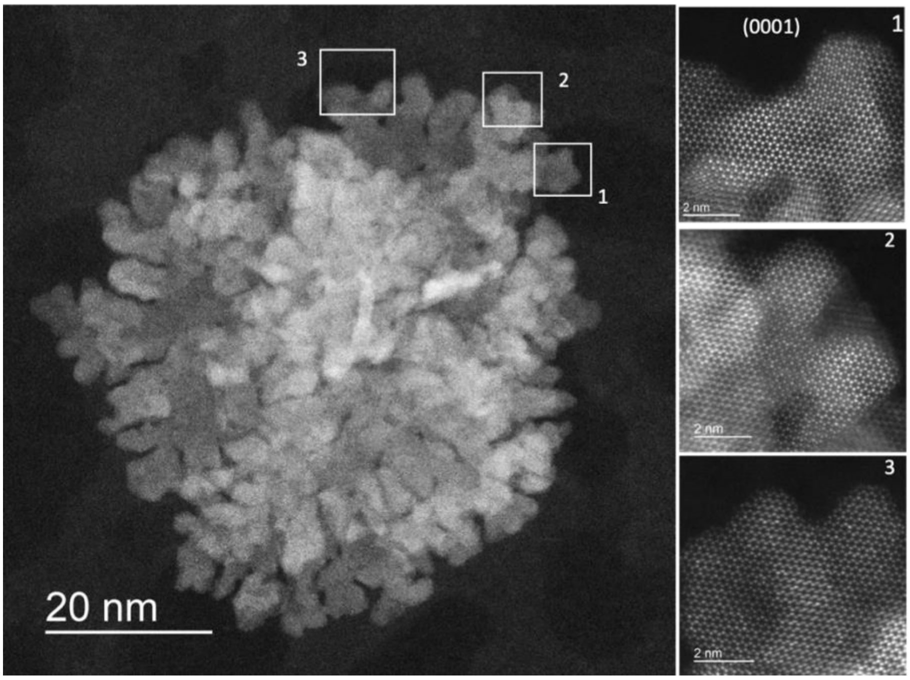

Fig. 2. Atomic-resolution HAADF-STEM images of RuIr-NC in multiple areas.

The exposed hcp (0001) plane can be seen in all three areas.

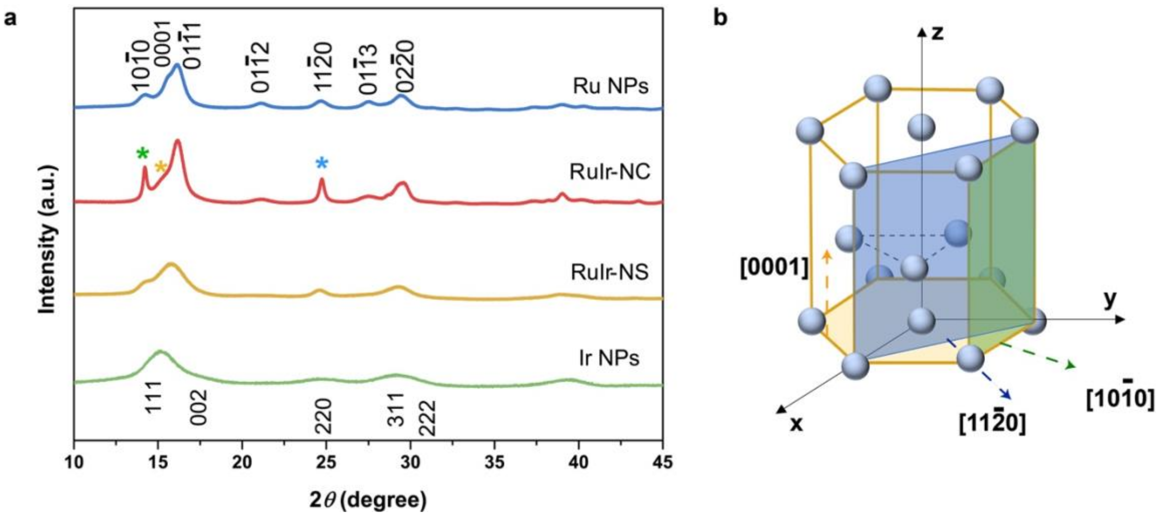

Fig. 3 Synchrotron powder XRD characterization of the prepared catalysts. a, XRD patterns of Ru NPs, **RuIr-NC**, **RuIr-NS**, and Ir NPs at 303 K, 2θ = 10 to 45°. The radiation wavelength was 0.580940 (1) Å. **b,** Schematic representation of the (01$\bar{1}$0) (green), (11$\bar{2}$0) (blue), and (0001) (orange) planes of an hcp structure. The colour of these planes is the same as the colour of the stars in **(a)**. The dashed arrows represent the normal direction of these planes, which are given by square brackets.

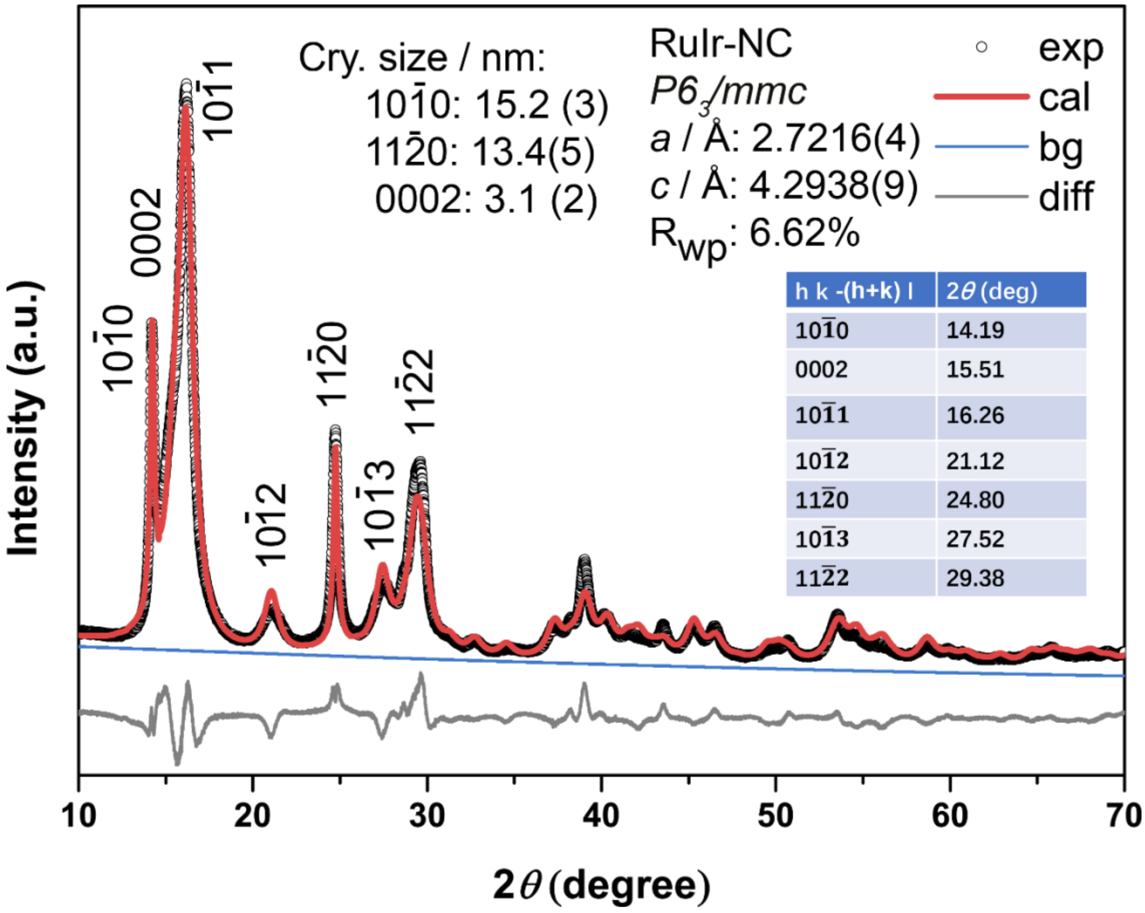

Fig. 4 XRD patterns and Rietveld refinement of RuIr-NC. The black circles are the experimental results. The red line is the calculated pattern. The bottom lines show the difference profile (grey) and the background item (light-blue). Crystal sizes obtained from different orientations are given in the inset presenting the extended (0001) plane in **RuIr-NC**. The table shows the Bragg positions.

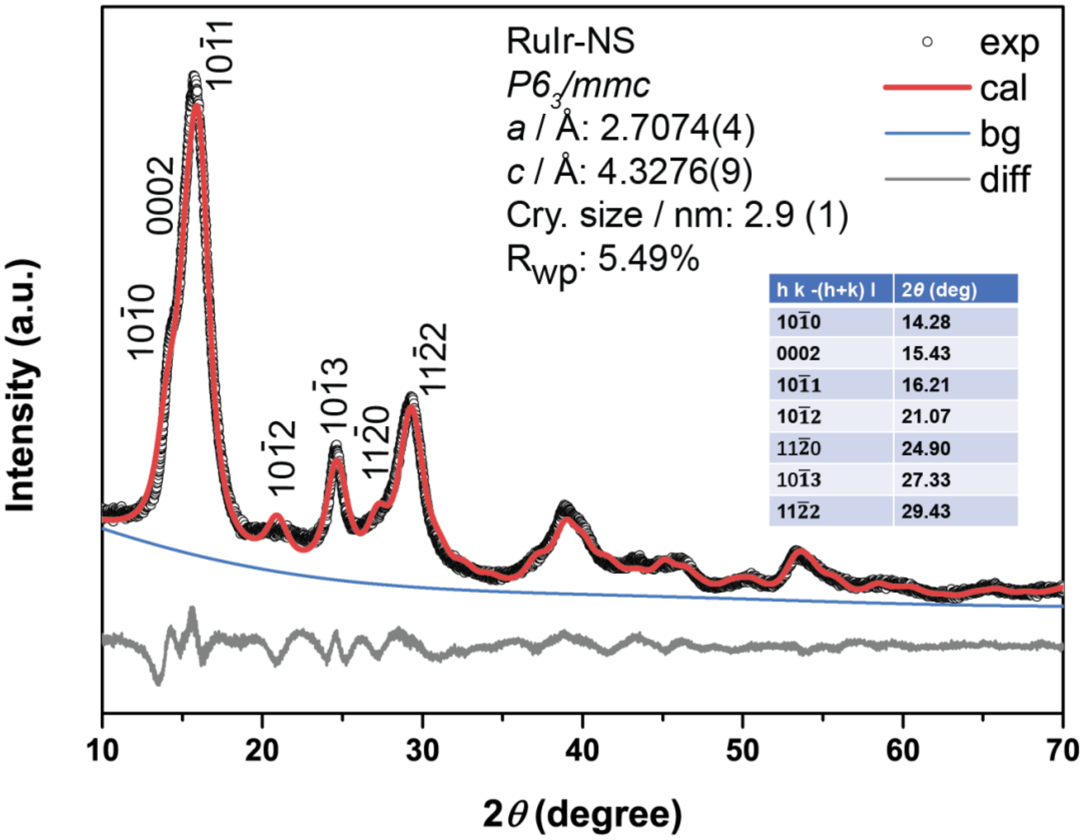

Fig. 5 XRD patterns and Rietveld refinement of RuIr-NS. The black circles are the experimental results. The red line is the calculated pattern. The bottom lines show the difference profile (grey) and the background item (light-blue). The table shows the Bragg positions.

Ru and Ir have very similar lattice parameter values ($a_{\text{Ru-fcc}}$ = 3.8233 Å vs. $a_{\text{Ir-fcc}}$ = 3.8312 Å). Therefore, it is very difficult to determine the formation of a solid solution between Ru and Ir only by the lattice parameter of **RuIr-NS** obtained by Rietveld refinement, particularly in nanoparticles with a diameter of less than 5 nm. However, within an allowable error, the obtained crystal size is reasonable.

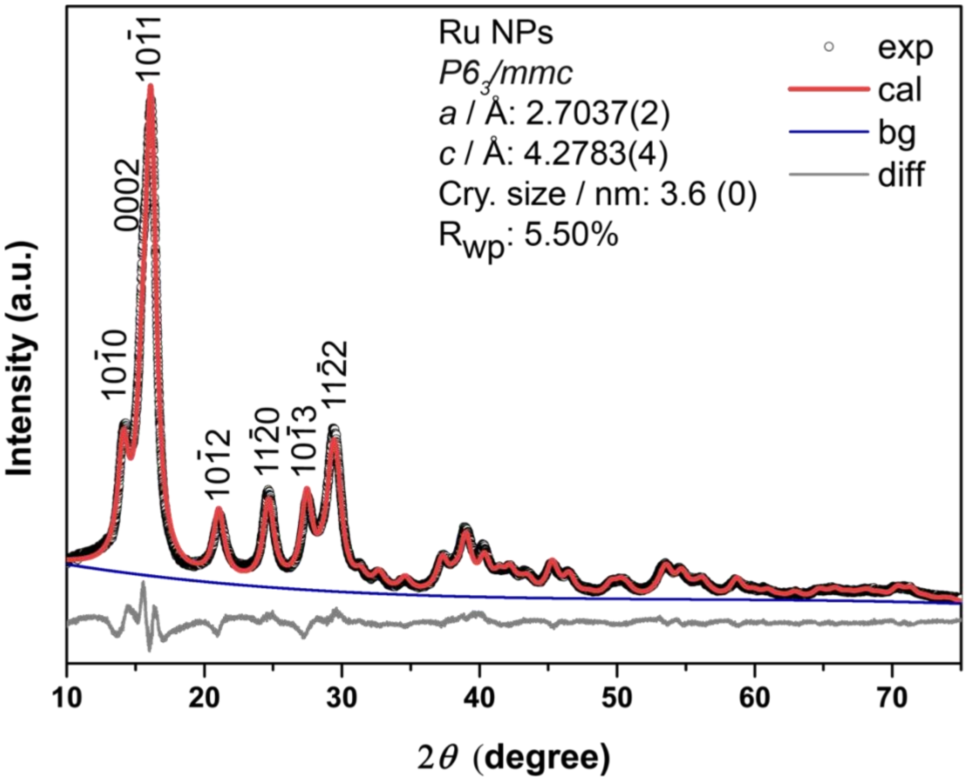

Fig. 6 XRD patterns and Rietveld refinement of Ru NPs. The black circles are the experimental results. The red line is the calculated pattern. The bottom lines show the difference profile (grey) and the background item (light-blue).

The lattice parameters of Ru NPs are well fitted with Ru bulk from the calculation (a = 2.70389 Å, c = 4.28168 Å). The obtained crystal size is consistent with the NPs' diameter obtained from TEM images.

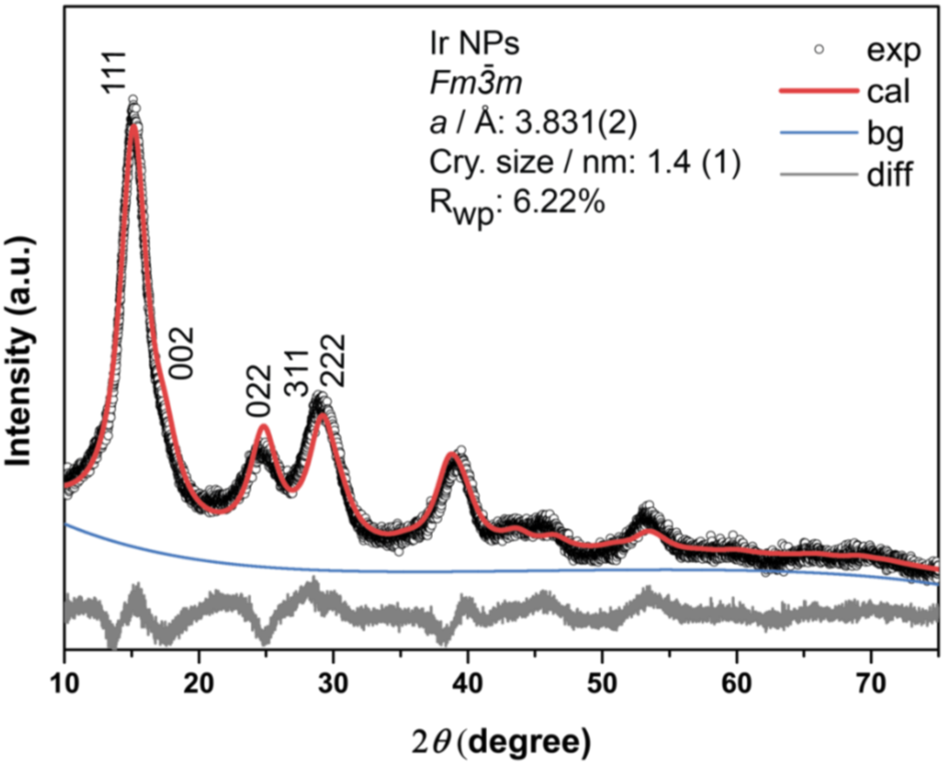

Fig. 7 XRD patterns and Rietveld refinement of Ir NPs. The black circles are the experimental results. The red line is the calculated pattern. The bottom lines show the difference profile (grey) and the background item (light-blue).

The lattice parameters of Ir NPs are well fitted with Ir bulk from the calculation (a = 3.8312 Å). The obtained crystal size is consistent with the NPs' diameter obtained from TEM images.

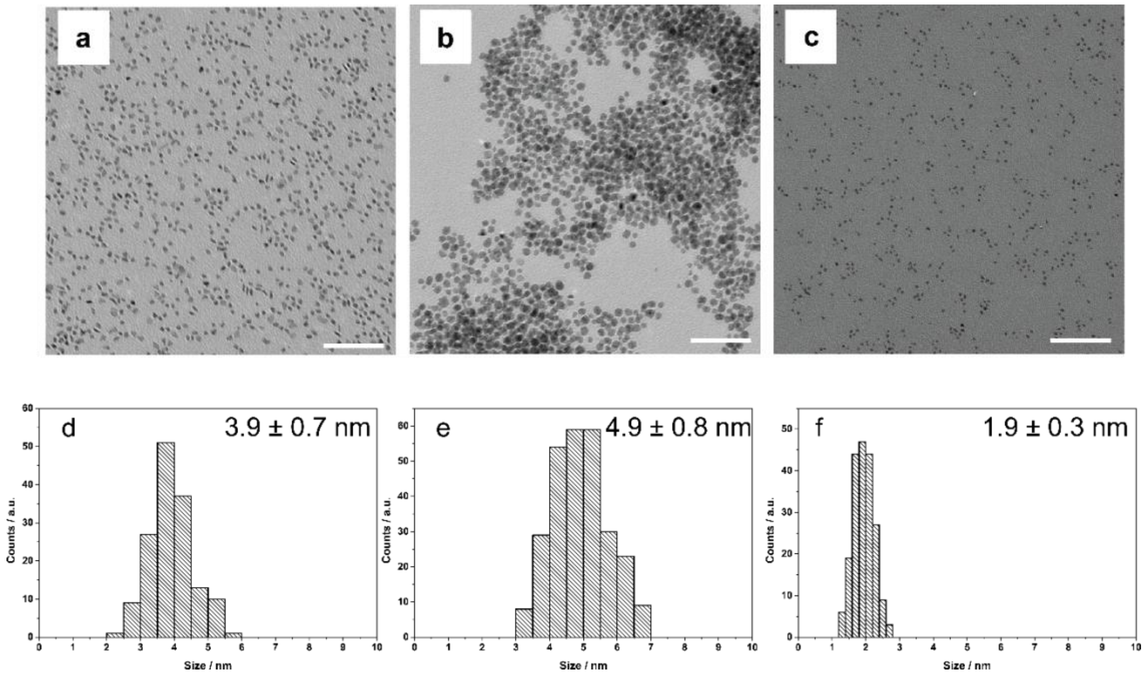

Fig. 8 Large-area, **bright-field TEM images of synthesized nanoparticles. a, RuIr-NS**. **b,** Ru NPs, and **c,** Ir NPs. **d-f**, the corresponding histogram showing the size distribution of a-c), respectively. The scale bar is 50 nm.

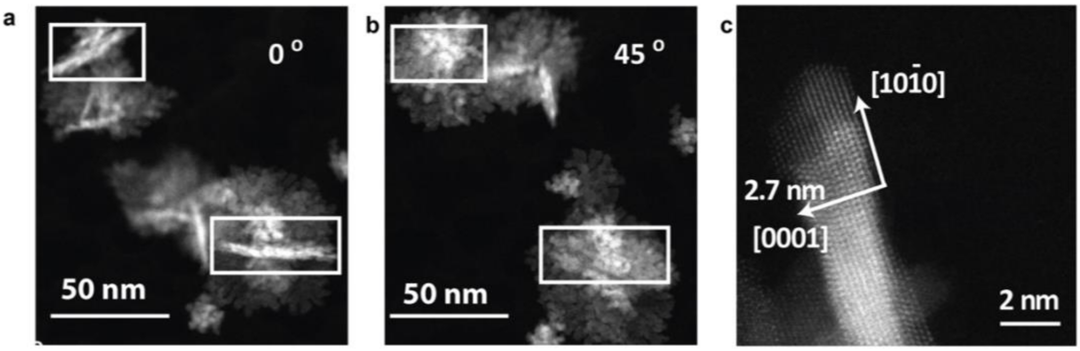

Fig. 9 STEM images of RuIr-NC in different view directions. a, At the zero-position, the white rectangles show a 1D bar-like shape. **b,** Tilting the position of **(a)** by 45 °. The bar changes into a coral-like shape. **c,** Atomic-resolution HAADF-STEM image showing the lateral view of a nanosheet. It grows along with the [10$\bar{1}$0] direction with a width of ca. 2.7 nm along with the [0001] direction, suggesting that a nanosheet comprises fewer than 10 stacked atomic layers.

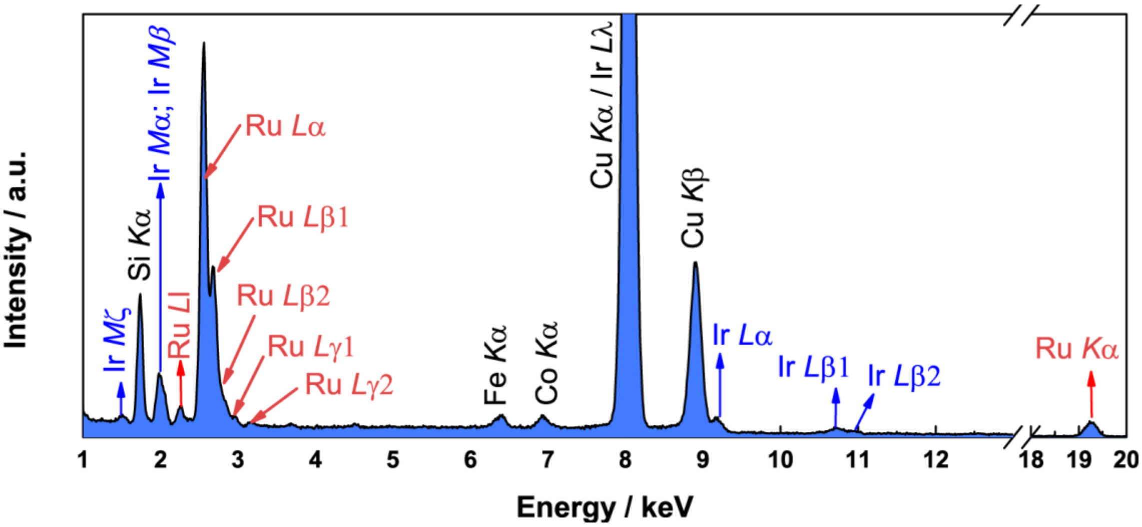

Fig. 10 EDS spectrum of the particle shown in Fig.1b. Clear Ru and Ir peaks were shown. Other signals were system peaks from the STEM and its grid.

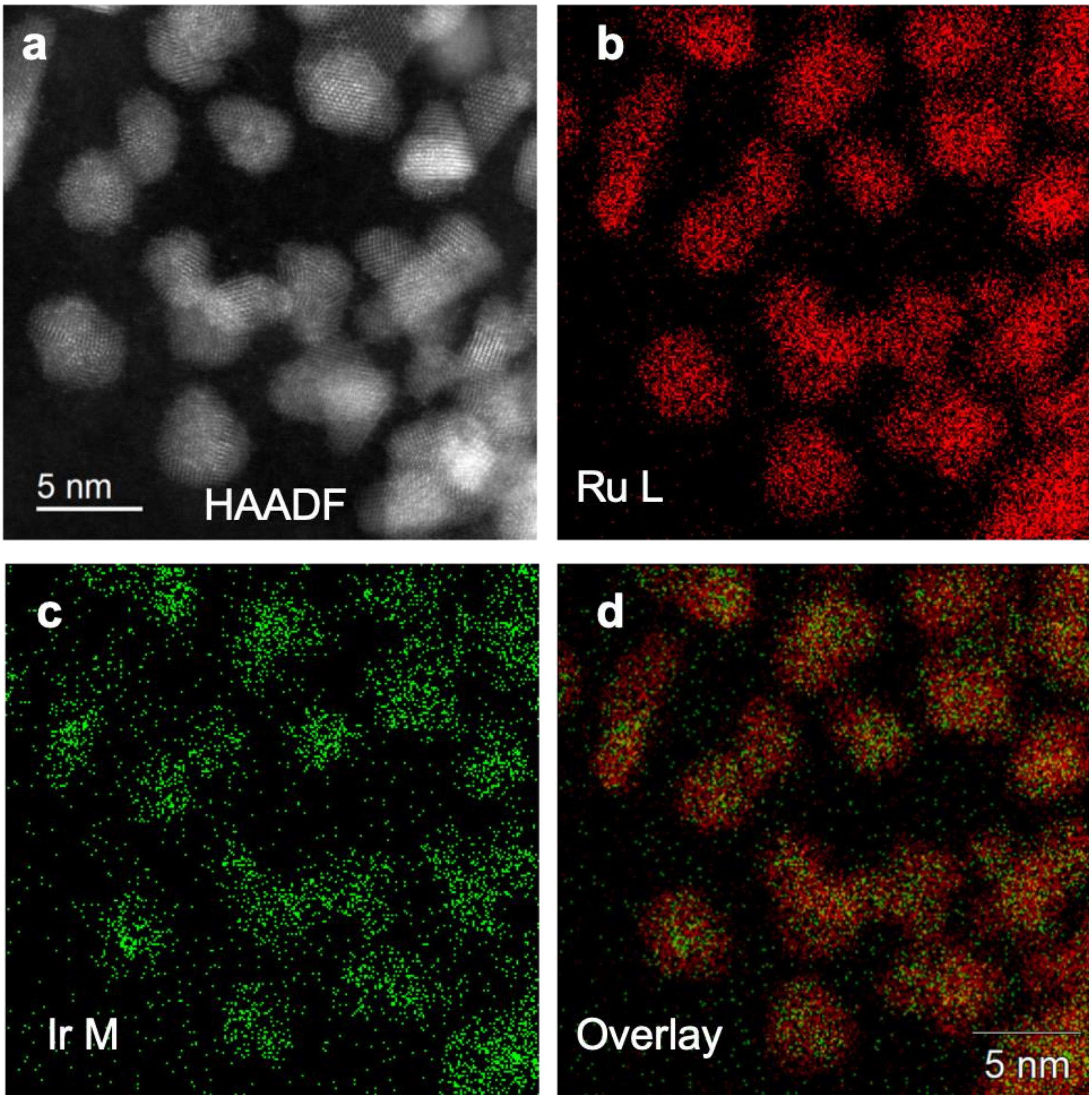

Fig. 11 Morphological and structural characterization of RuIr-NS NPs by HAADF-STEM. a, HAADF image and the corresponding elemental mapping showing **b,** Ru (red), **c,** Ir (green), and **d,** overlay.

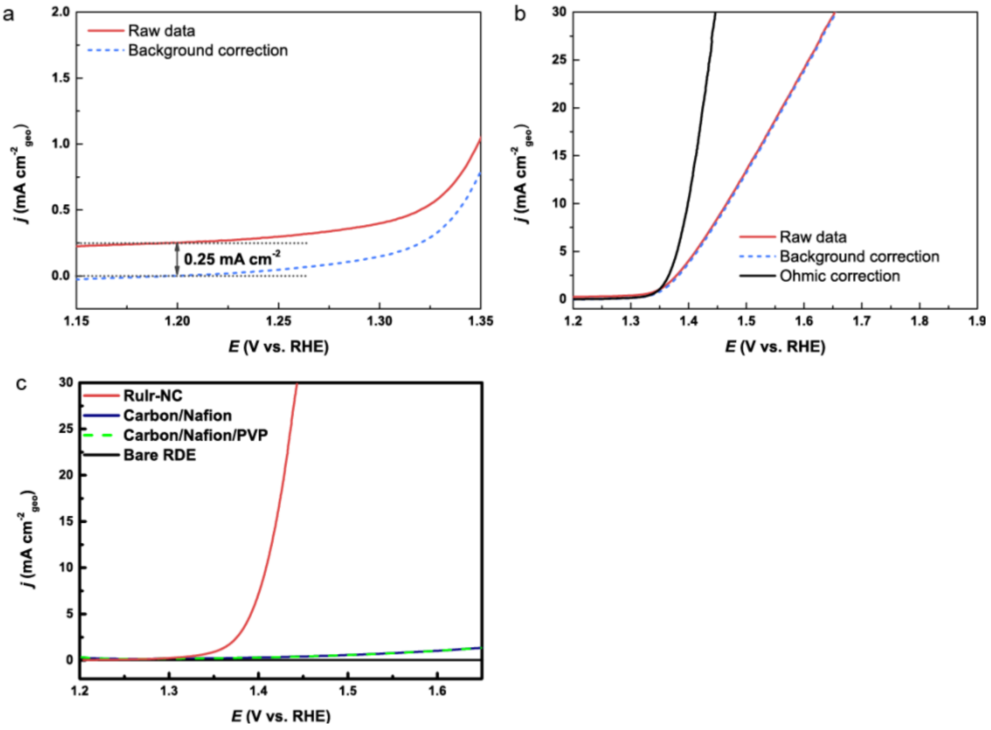

Fig. 12 OER background and Ohmic corrections of the OER activity of RuIr-NC. **a**, polarization curve of **RuIr-NC** ("Raw data", solid red line, shown in the range of 1.15 — 1.35 V) was corrected by subtracting the background current density. The current density at 1.2 V, 0.25 mA cm^{-2}, is considered as the background current density which comes from the capacitance current and oxidation of metal and carbon before OER. With the background-correction, the current is almost zero before the onset of OER (dashed blue line). **b,** the ohmic-corrected OER current (dashed blue line) is then corrected with the measured resistance (≈38.3 Ω) to yield the final electrode OER activity (solid black line). **c,** comparing the LSVs of **RuIr-NC**, bare RDE electrode and carbon (Vulcan 72R)/Nafion mixture. At 1.46 V the current density of **RuIr-NC** is two and four orders of magnitude of those of carbon/Nafion mixture and RDE, respectively. Therefore, the current contributed from these backgrounds at high potentials are negligible. Test condition: 0.05 M H_2SO_4, 1600 rpm, 5 mV s^{-1}.

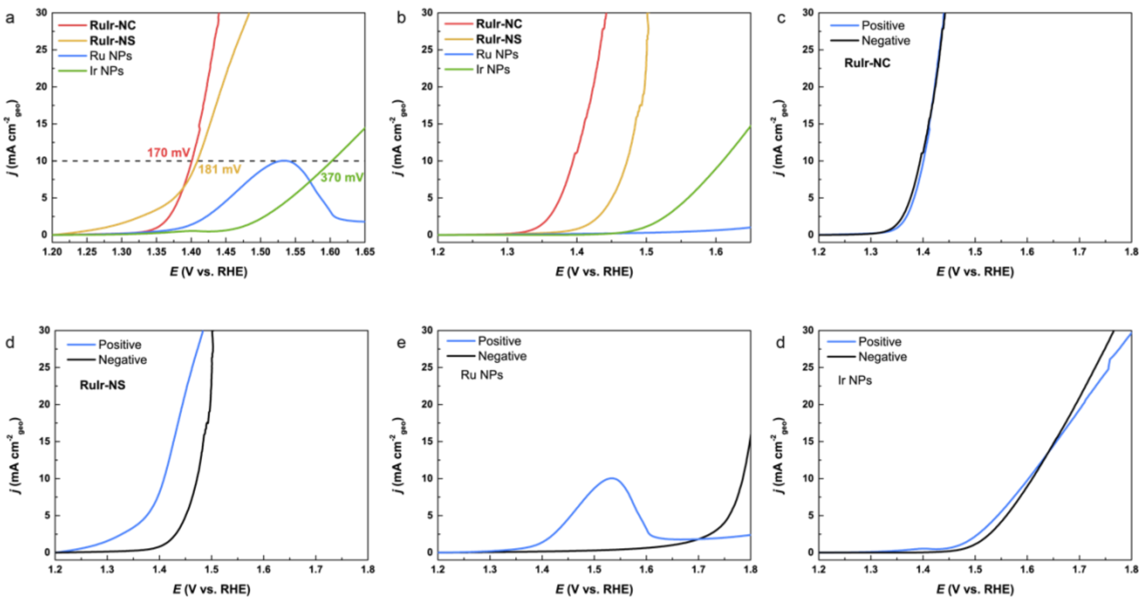

Fig. 13 OER performance in O_2-saturated 0.05 M H_2SO_4. a, LSVs of the tested catalysts obtained with positive scan direction (from high potential to low potential). **b,** LSVs of the tested catalysts obtained with negative scan direction (from high potential to low potential, same as Fig. 2a in the main text). **c-f**, comparison of the LSVs obtained by positive (blue line) and negative (black line) scan direction.

For **RuIr-NC**, there is little difference between positive and negative LSVs, which also supports our conclusion that **RuIr-NC** has high stability and activity.

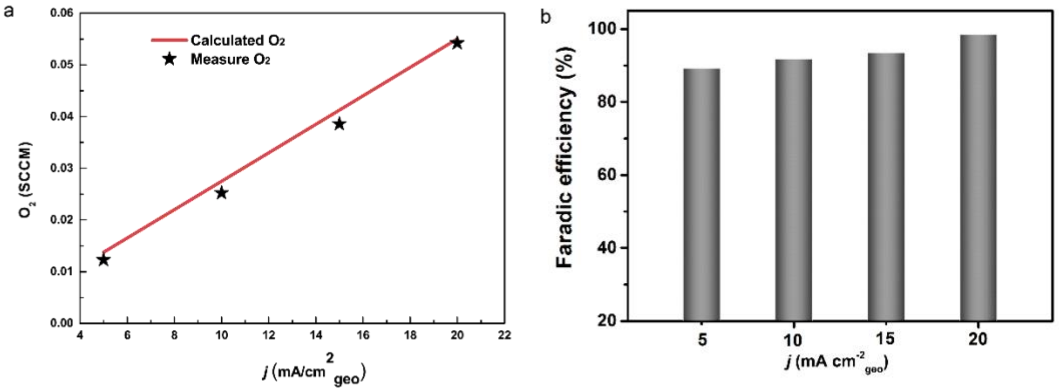

Fig. 14 Faradic efficiency of RuIr-NC. a, calculated O_2 and measured O_2 amount by GC. b, Faradaic efficiency of **RuIr-NC**. Please note that the current density is not capacitance corrected, therefore the loss of faradic efficiency at a lower current density should mainly come from the oxidation of metal and carbon/PVP/Nafion.

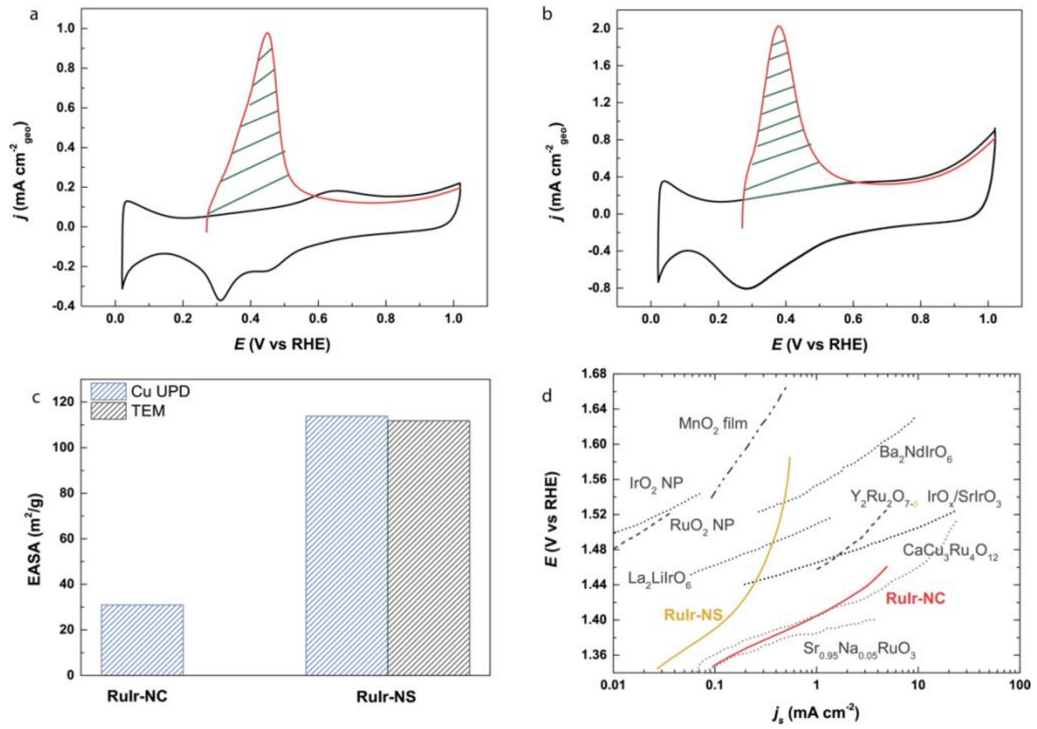

Fig. 15 Specific activity (j_S) based on electrochemistry active surface area (EASA). Illustration of the EASA evaluation method based on Cu UPD for **a, RuIr-NC** and **b**, **RuIr-NS**. **c**, the obtained EASAs. The EASA based on TEM is only valid on a spherical model (Ref. *55*, *56*). **d**, specific activity (j_S) based on the EASA values together with the high-performance catalysts with considerable stability in acid including MnO_2[10], IrO_2 and RuO_2 NP[11], La_2LiIrO_6[12], $BaNdIrO_6$[13], $Y_2Ru_2O_{7-\delta}$[2], $CaCu_3Ru_4O_{12}$[14], $Sr_{0.95}Na_{0.05}RuO_3$[15], and $IrO_x/SrIrO_3$[16].

The EASA values of **RuIr-NS** obtained by two methods are quite similar (114 vs. 111 m^2/g, Supplementary Fig. 15c) which verified the accuracy of EASA obtained by Cu UPD. The ECSA of **RuIr-NC** is 31.0 m^2/g.

Although **RuIr-NC** has a smaller surface area than **RuIr-NS**, it shows very similar OER activity toward **RuIr-NS** below 1.4 V and better stability. These results exclude the influence of the surface area on the OER performance, at least on the OER stability.

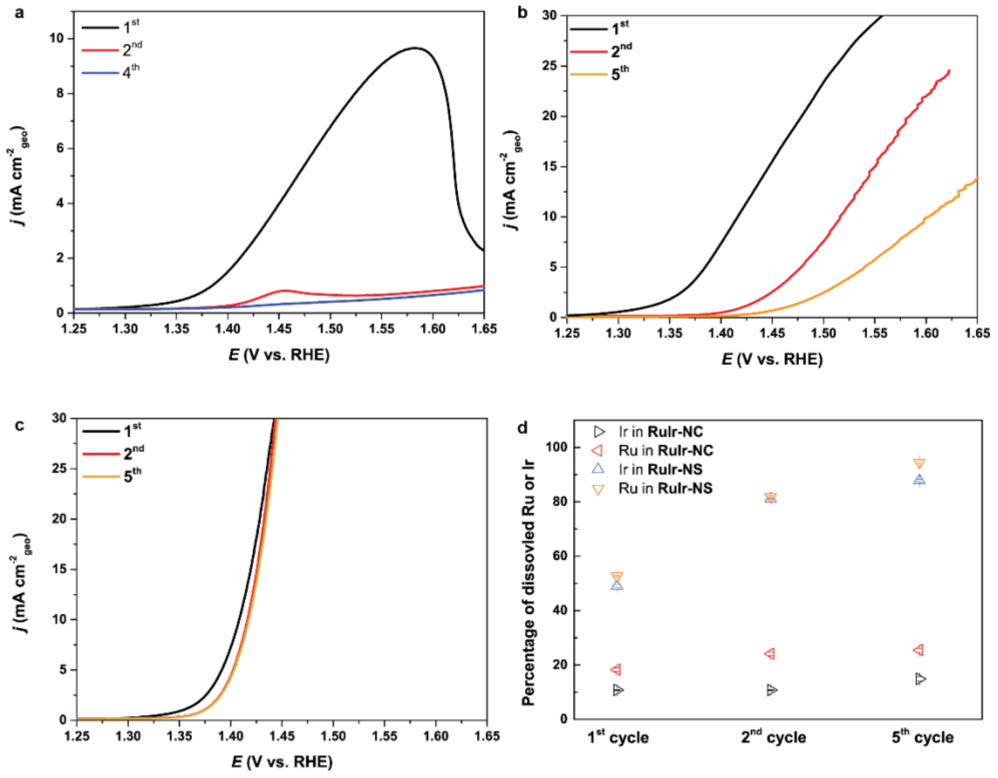

Fig. 16 Continuous OER polarization curves of catalysts and ICP analysis. a. Ru NPs. **b**, **RuIr-NS**. **c**, **RuIr-NC**. **d**. ICP results show the dissolved percentage of Ru or Ir in the electrolyte after the 1st, 2nd, and 5th LSV scan. Error bars were obtained by three independent ICP tests. The weight percentages were obtained by dividing the dissolved amount by the total amount on the electrode before OER. LSV scan condition: 5 mV s^{-1}, 1600 rpm. Scan direction: anodic. The time of the CP test for **RuIr-NS** and **RuIr-NC** is 50 min and 122 h, respectively. Please note the Ir percentages during LSV scans in **RuIr-NC** might not so accurate due to the low amount.

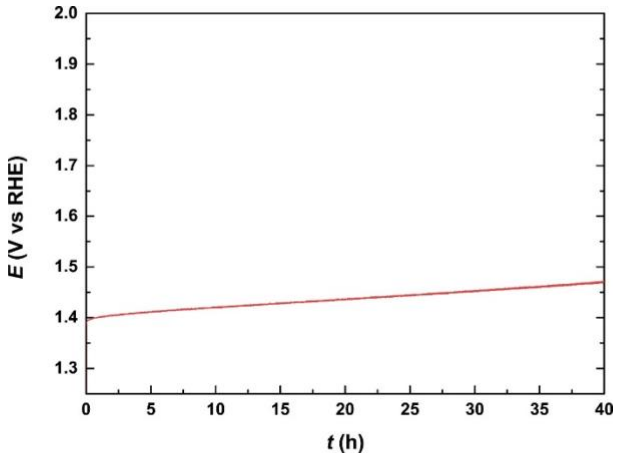

Fig. 17 CP curve of RuIr-NC at the OER current density of 10 mA cm^{-2}$_{geo}$. The **RuIr-NC** can sustain its activity for more than 40 h (with less than 5% increase of the potential).

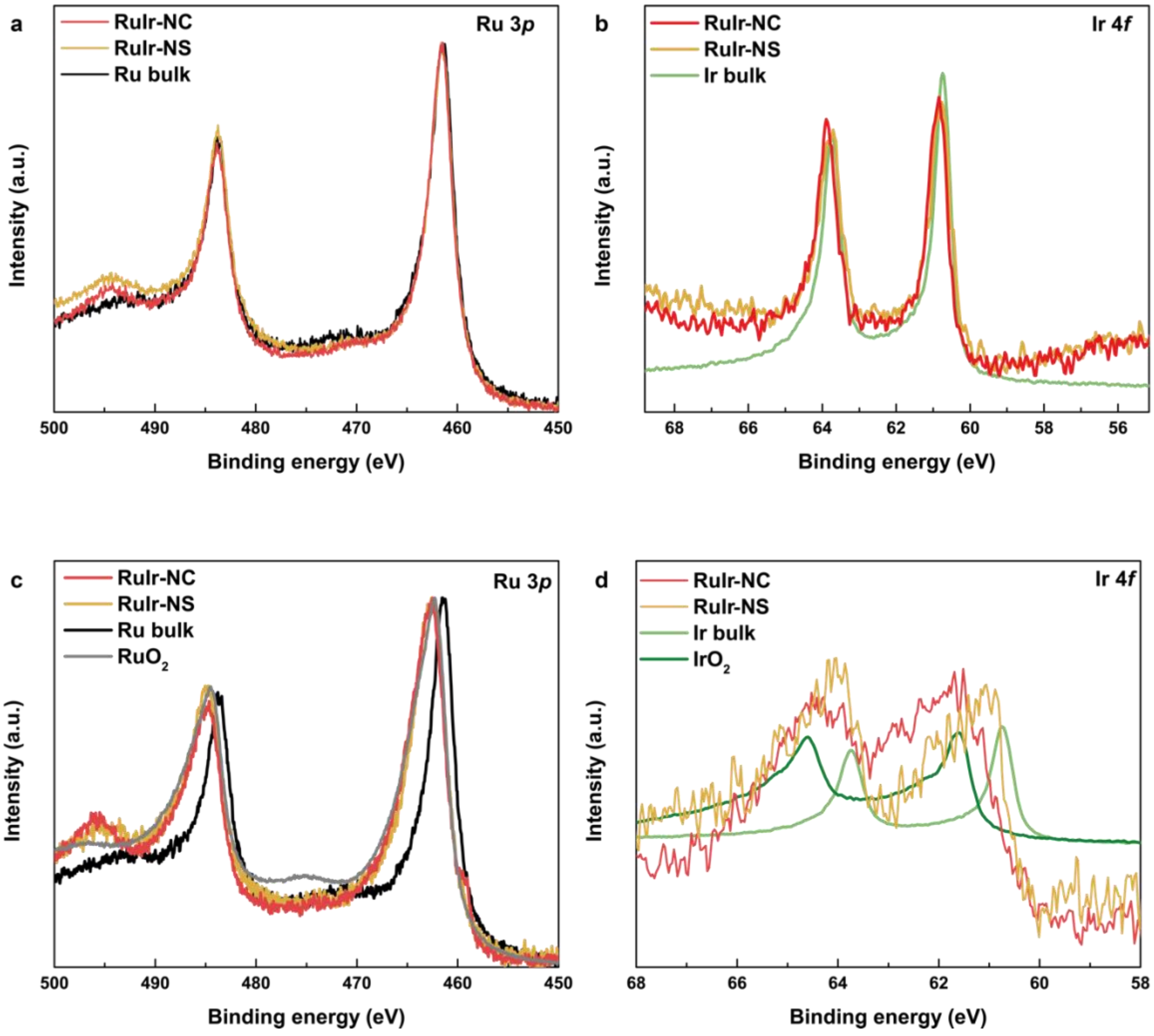

Fig. 18 HAXPES core levels of RuIr catalysts before (a, b) and after (c, d) operando XANES. (**a, c**) Ru 3*p*. (**b, d**) Ir 4*f*.

Before OER, the Ru 3*p* spectrum of **RuIr-NC** overlaps with that of **RuIr-NS** as well as that of Ru bulk, suggesting the same metallic nature. After operando XANES, the Ru3p spectrum of both RuIr samples shifts to the position of RuO_2, which is consistent with the XANES results.

Regarding Ir 4*f*, before OER, RuIr samples have overlapping Ir 4f spectra, suggesting a similar Ir effect on both samples. The peak position, however, shifts to higher energy by 0.1 eV than Ir bulk, suggesting an electronic structure change in the Ir sites that are surrounded by Ru atoms. After operando XANES, although Ir 4*f* spectra become noise, the obvious shift to the IrO_2 side demonstrates the oxidation of Ir.

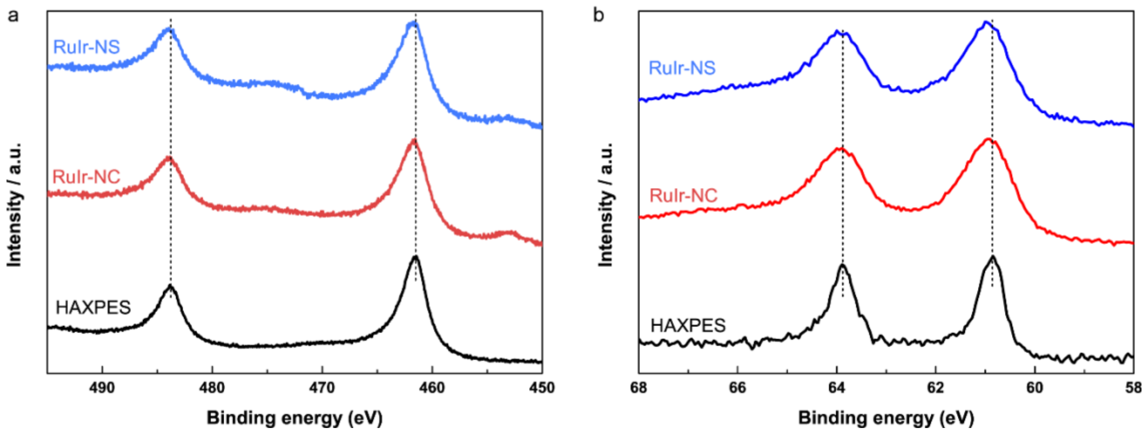

Fig. 19 Comparison of core level spectra obtained by lab XPS and HAXPES. a, Ru3*p* spectra. **b**, Ir4*f* spectra.

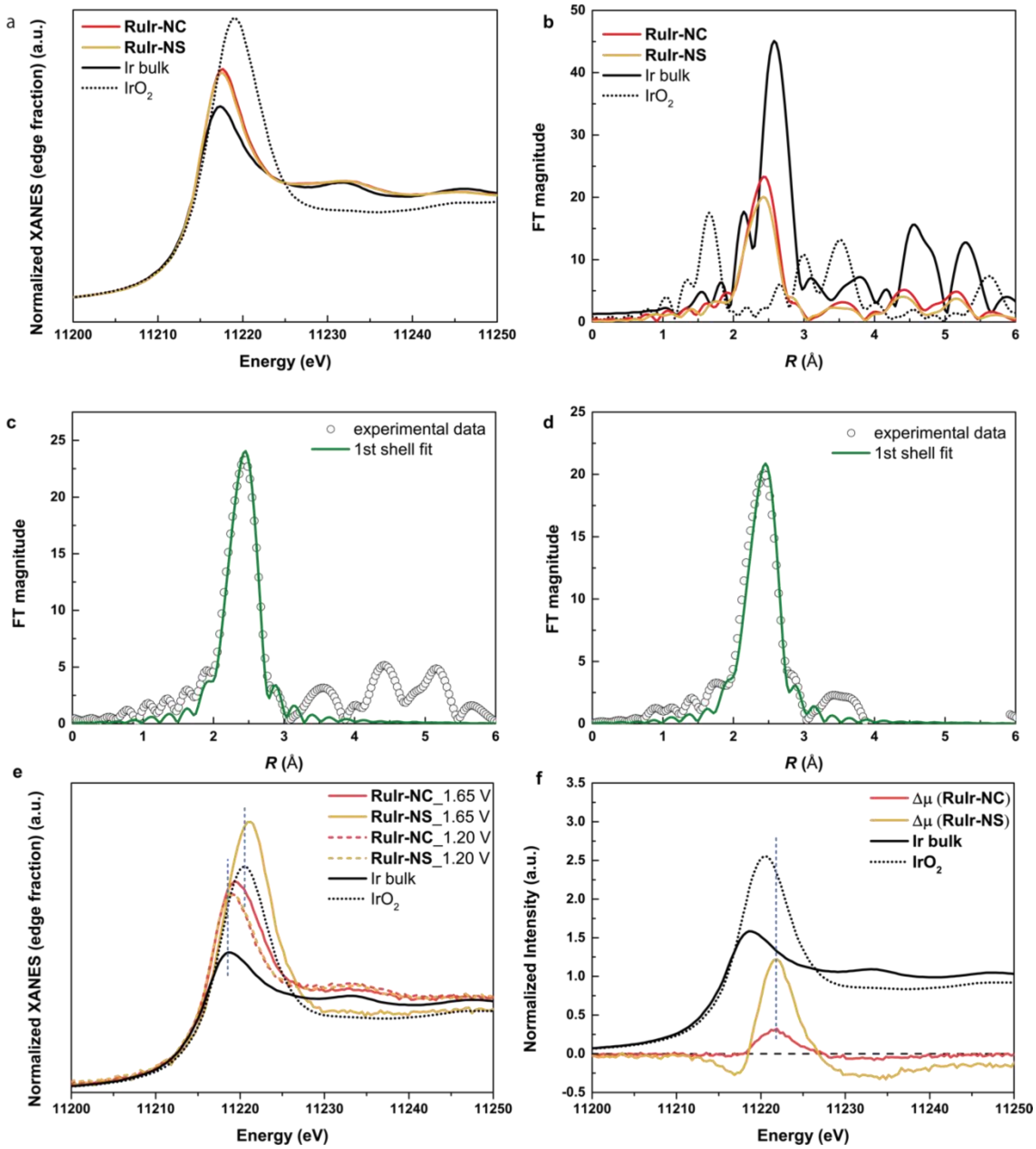

Fig. 20 Local structure of Ir sites. a, Ir *L*3-edge XANES spectra of the as-prepared RuIr catalysts. **b,** Ir *L*3-edge EXAFS spectra. The first-shell fitting of **c, RuIr-NC** and **d, RuIr-NS. e,** Ir *L*3-edge XANES spectra of the as-prepared RuIr catalysts at 1.20 V and 1.65 V along with Ir and IrO_2 as a comparison. **f,** Ir *L*3 $\triangle\mu$ ($\mu_{1.65\,V}$ - $\mu_{1.20\,V}$) spectra. **a-d** and **e** are the as-prepared samples and samples during OER, respectively.

Fig. 20a-d suggests that the local structures of Ir in the as-prepared **RuIr-NC** and **RuIr-NS** are quite similar. Therefore, to account for the different catalytic performances between **RuIr-NC** and **RuIr-NS**, we can exclude the contribution from Ir.

Fig. 20e shows the operando *L*3-edge XANES spectra of RuIr catalyst during OER. It is known that the melic Ir changes to Ir^{4+} in the range of 0.6-1.0 V, a potential lower than the OER thermal-equilibrium potential (1.23 V). [17] Therefore, Ir^{4+} species might already exist in RuIr catalysts after CV cleaning. To verify this, firstly, we measured the XANES of RuIr catalysts at 1.20 V (after CV cleaning) (dotted red and yellow lines in Fig. 20e). The white line (WL) positions of these spectra are located at lower energy than that of IrO_2. This is because the inherent bulk average nature of XAFS experiments. The contribution of the metallic core dominated over the amorphous surface at 1.2 V. Next, XANES spectra of the two catalysts at 1.65 V were measured. We found that the XANES at 1.65 V changes obviously compared to the ones at 1.2 V. To understand the change at the surface, we used a delta mu ($\triangle\mu$) method to isolate surface change by subtracting out the bulk information. The WL position of $\triangle\mu$ spectra of both RuIr catalysts is 1.3 V higher than the IrO_2. As for Ir L_3-edge, the increase of *d*-band hole is a function of the positive shift of WL position, that is 0.9 - 1.0 eV per *d*-band hole, which has been confirmed elsewhere.[18, 19] With higher oxidation states (> 6+), this value might be slightly larger considering the covalence of bond. Therefore, the 1.3 eV shift could imply the existence of higher oxidation states (>4+) of Ir in the RuIr catalysts because Ir^{4+} species already exist in the surface of RuIr catalysts at 1.2 V.

We note that the oxidation of Ir in **RuIr-NS** is faster than that in **RuIr-NC**, which is consistent with the change of Ru *K*-edge.

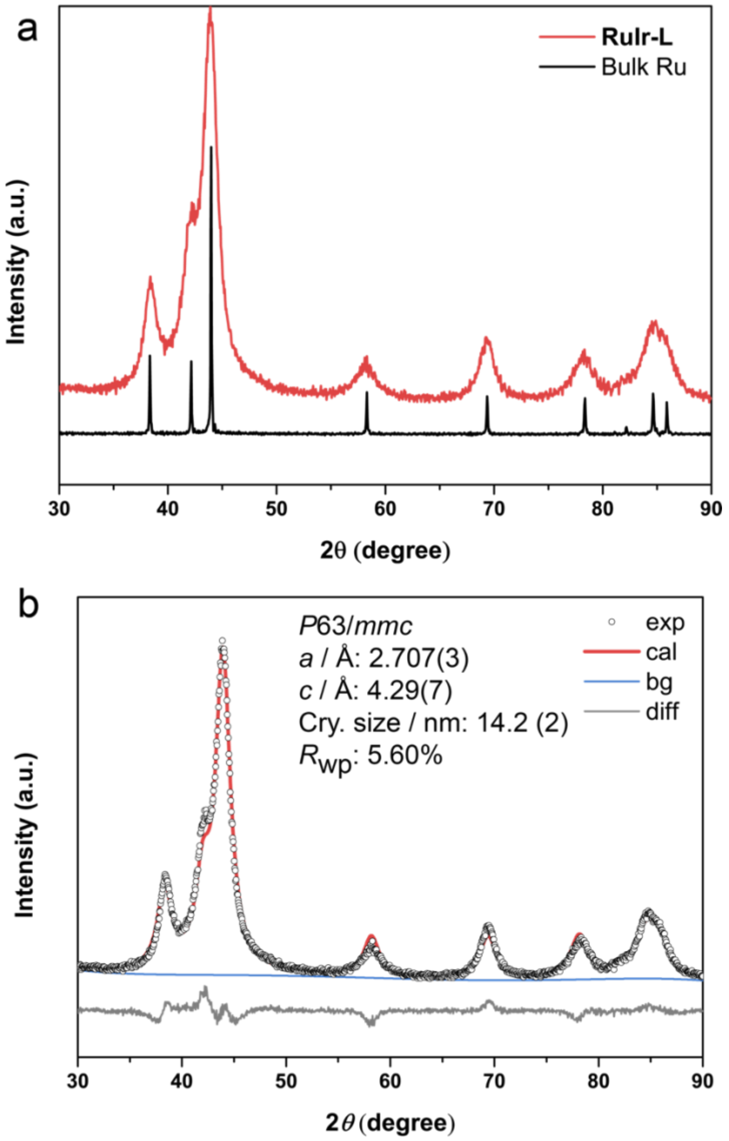

Fig. 21 XRD characterization of RuIr-L. **a,** XRD pattern of **RuIr-L**, showing the same hcp structure as that of Ru bulk, which is isotopically grown Ru–Ir NPs. **b,** Rietveld refinement of **RuIr-L**. The black circles are the experimental results. The red line is the calculated pattern. The bottom lines show the difference profile (grey) and the background item (light-blue). The radiation wavelength was 1.54 Å.

The crystal size obtained from the Rietveld refinement is 14.2(2) nm, which is similar to that of **RuIr-NC**.

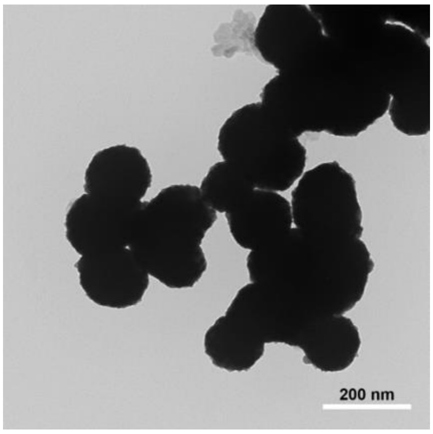

Fig. 22 TEM images of RuIr-L.

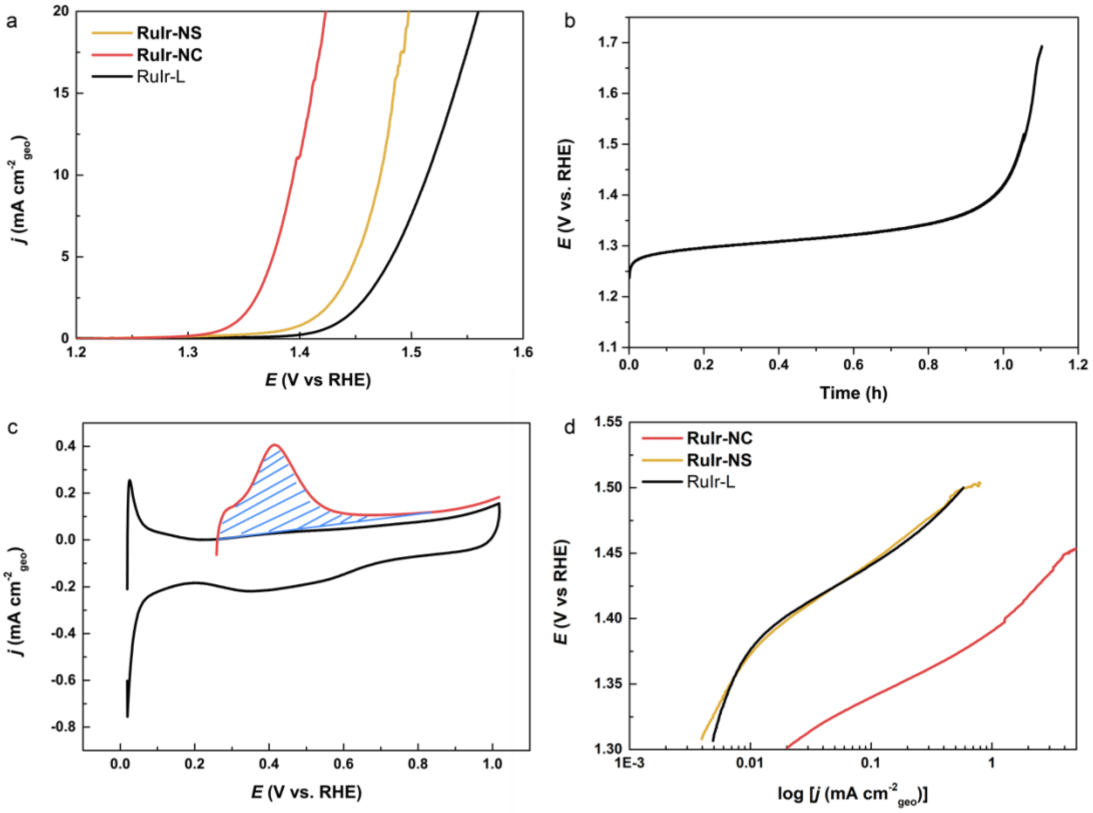

Fig. 23 OER performance of RuIr-L. a, the geometric activity of **RuIr-L**. **b,** Chronopotentiometric curves of **RuIr-L** at a current density of 1 mA/cm^{-2}. c, EASA evaluation of RuIr-L based on Cu UPD. d, the specific activity of **RuIr-L**. **RuIr-NC** and **RuIr-NS** were used for comparison. Scan direction: cathodic.

Although **RuIr-L** has a similar crystal size to that of **RuIr-NC**, **RuIr-L** requires a much higher overpotential to achieve 10 mA cm$^{-2}{}_{geo}$. Also, the CP result in Fig. 23b suggests much lower stability than that of **RuIr-NC**. Considering the specific activity, **RuIr-NC** is higher than both **RuIr-L** and **RuIr-NS**.

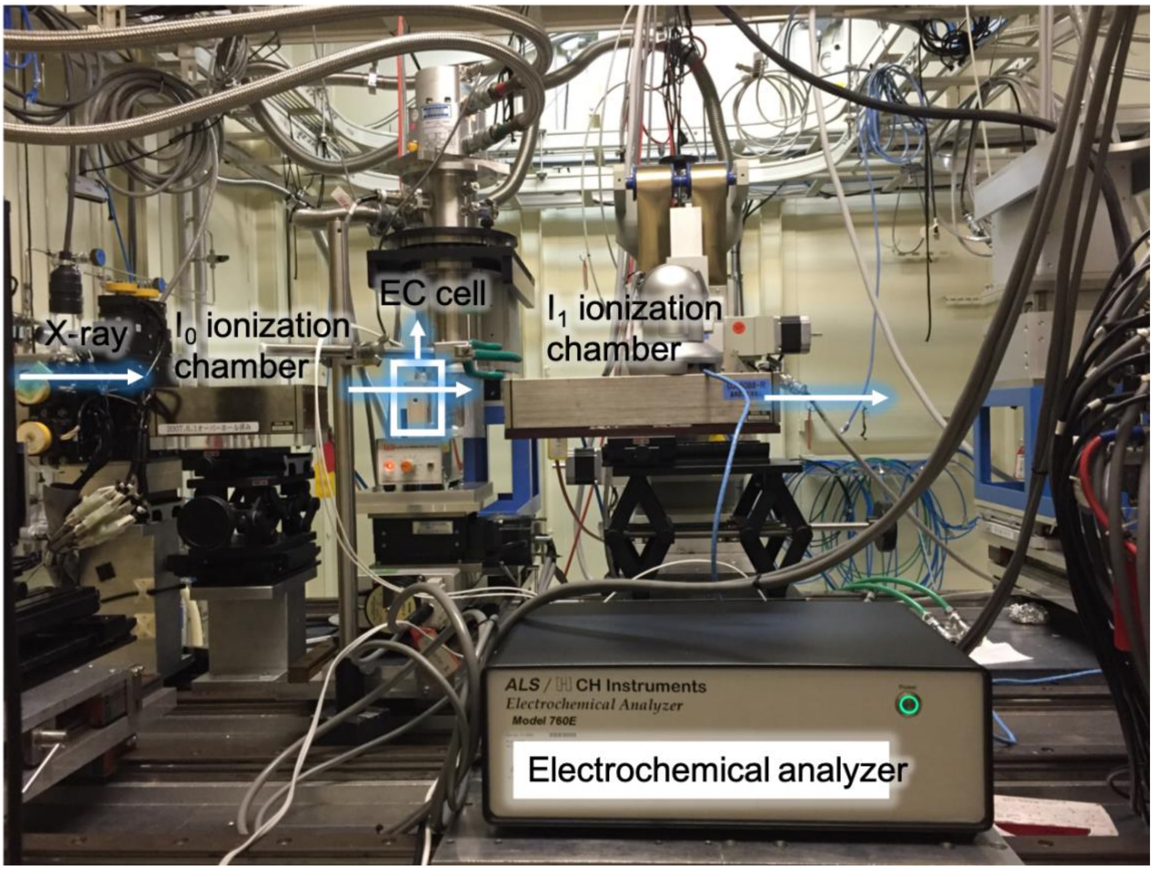

Fig. 24 Experimental setup for operando XAFS.

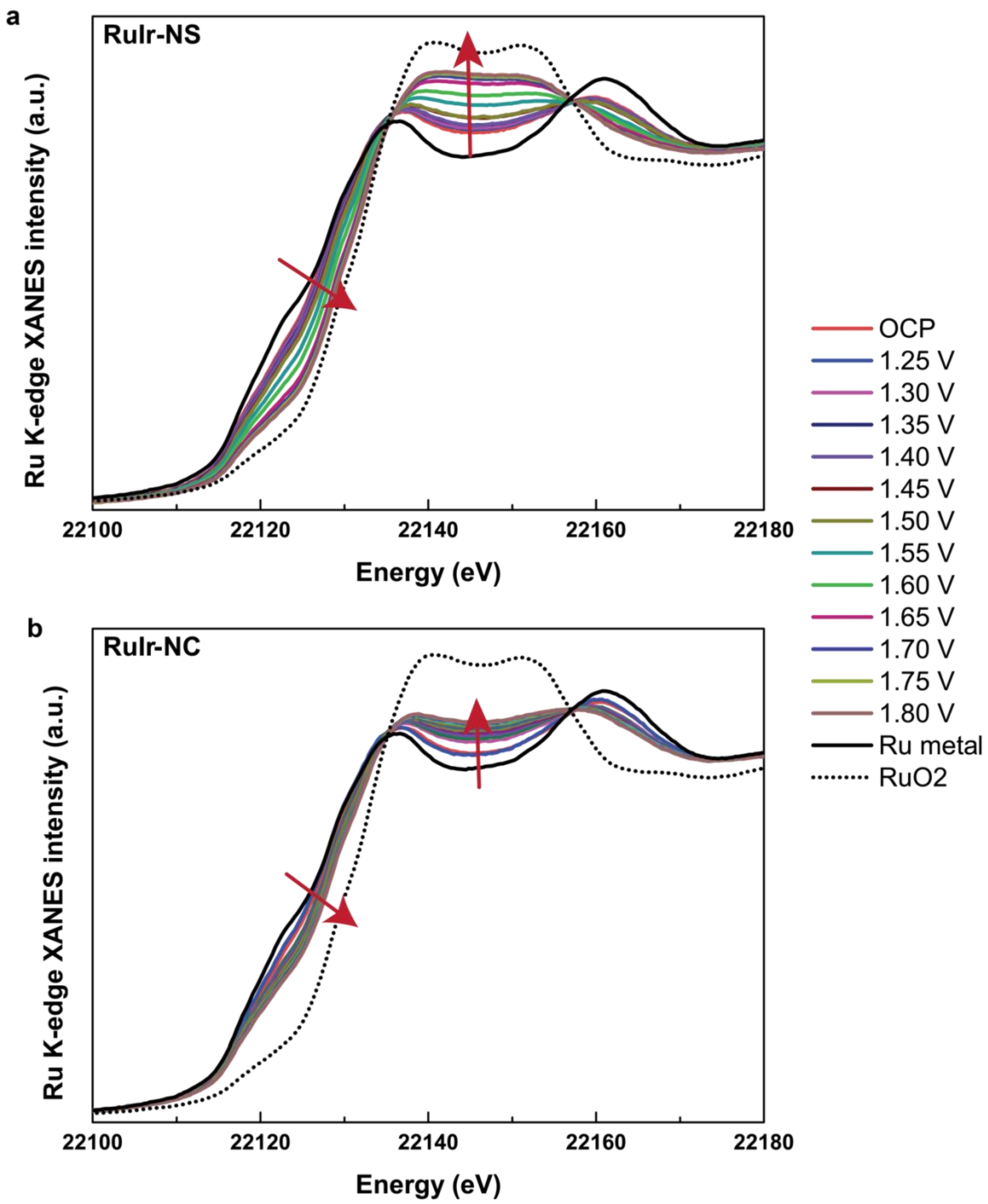

Fig. 25 Operando XANES on the Ru *K*-edge. a, RuIr-NS and **b, RuIr-NC**. The direction along the red arrows is from the OCP to 1.80 V.

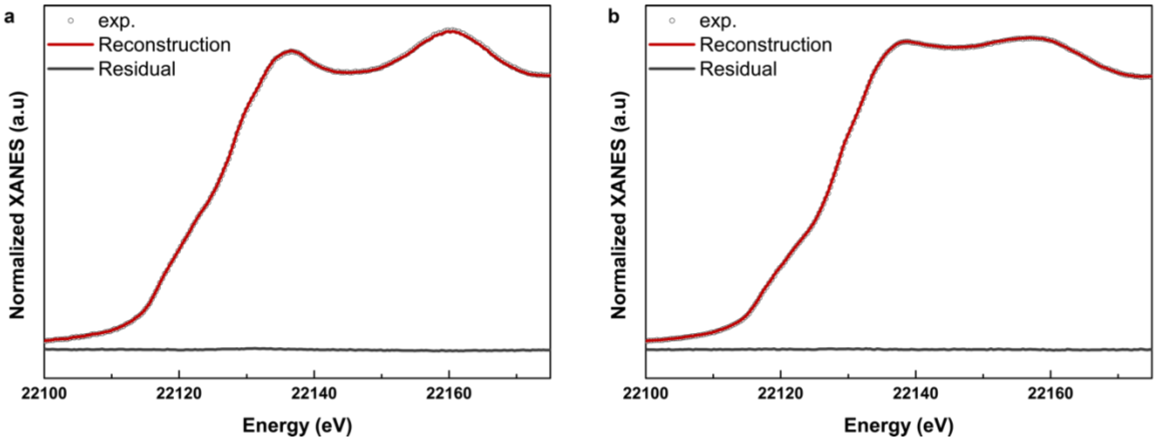

Fig. 26 Normalized XANES spectra of RuIr-NC reconstructed using two components. **a,** 1.25 V. **b,** 1.80 V.

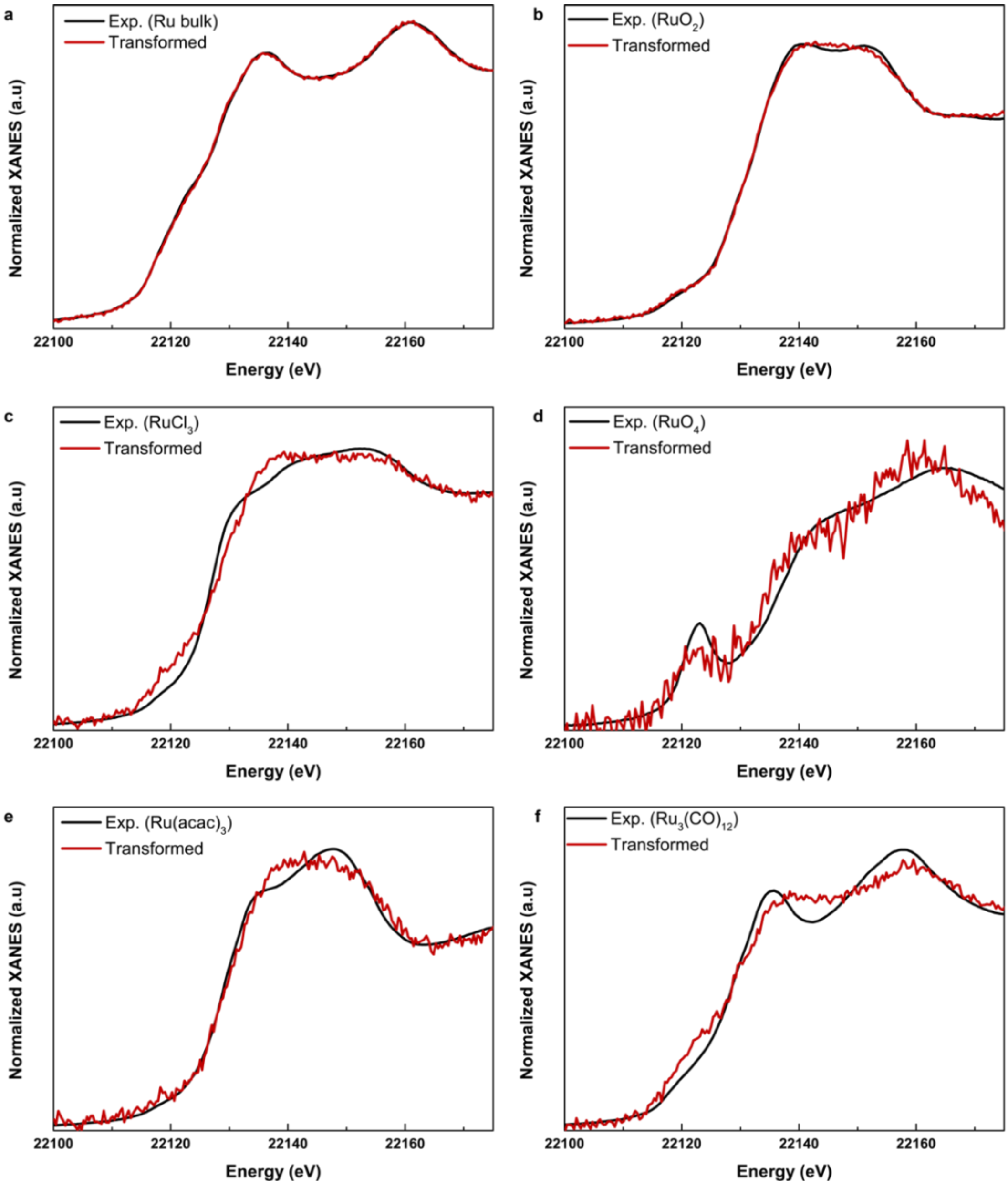

Fig. 27 Target transformation results (red lines) for standard references (solid lines). a, Ru bulk metal. **b**, RuO_2. **c,** $RuCl_3$. **d,** RuO_4. **e,** $Ru(acac)_3$. **f,** $Ru_3(CO)_{12}$. Ru and RuO_2 are found to yield a sufficient match upon transformation. The other four species that show unmatched transformed curves are likely not present in the normalized spectra.

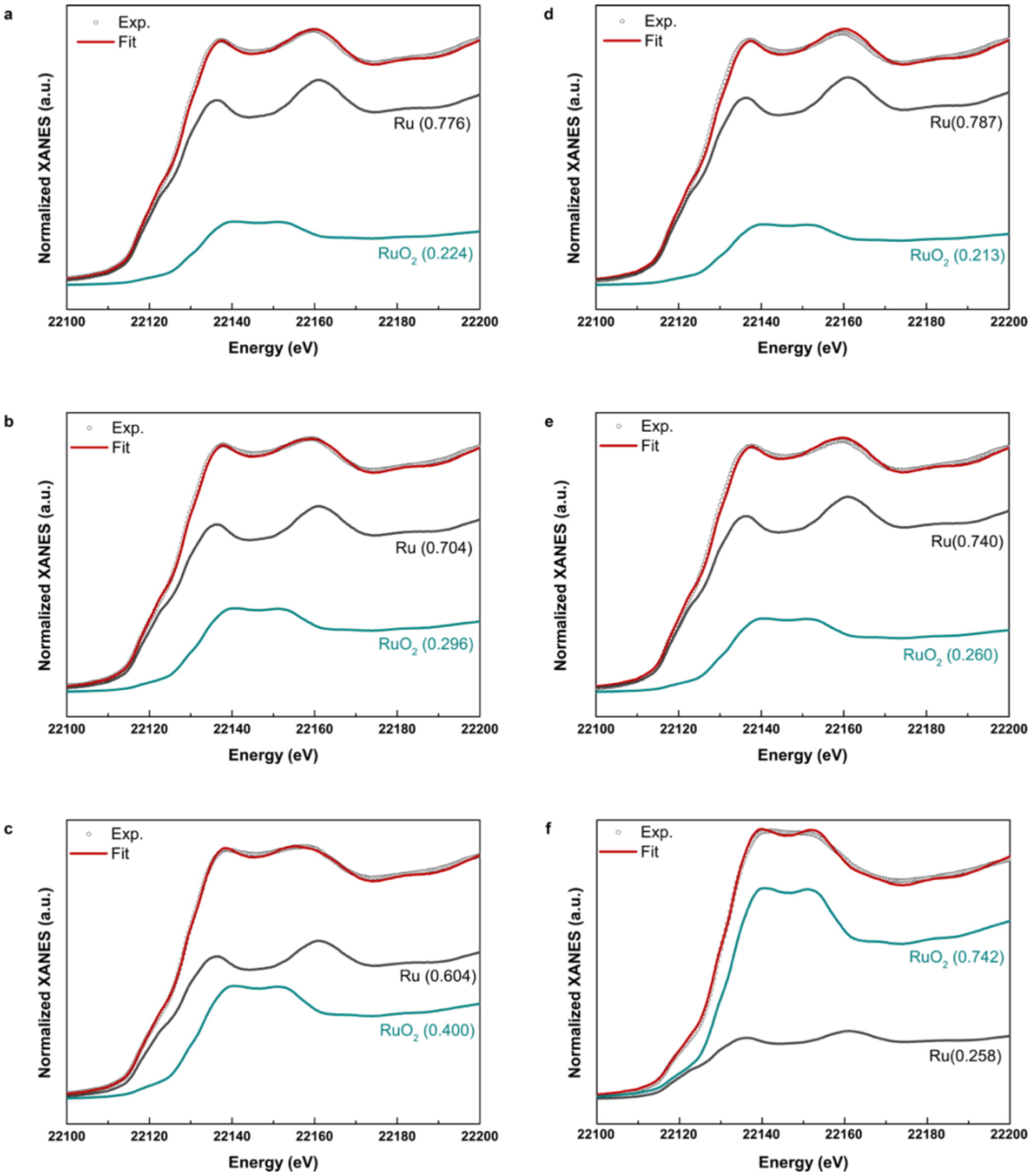

Fig. 28 Experimental results and LCF for the normalized XANES spectra of the Ru *K*-edge collected at different potentials. a–c, RuIr-NC. **d–f, RuIr-NS**. **a and c,** 1.25 V. **b and d,** 1.40 V. **c and f,** 1.80 V. The black circles and solid red line are the experimental data and fitting curve, respectively. The percentages of the two components (black and green solid lines)are also given. The detailed fitting results of all the sets of XANES spectra are shown in Table 2.

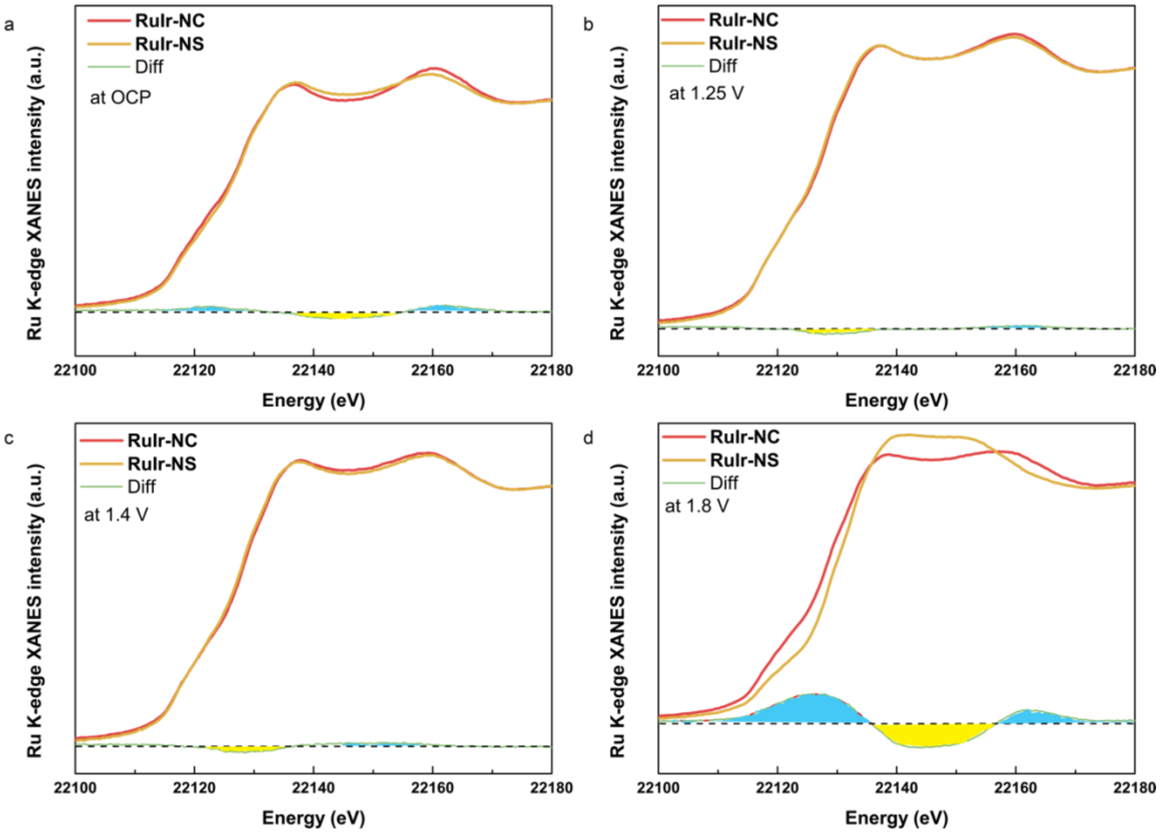

Fig. 29 Differences in XANES spectra between RuIr-NC and RuIr-NS in the range from −20 to 60 eV. At **a,** OCP, **b,**1.25 V, **c,** 1.40 V, and **d**, 1.80 V. The yellow and blue areas show a decrease and increase in the intensity of **RuIr-NC** compared with **RuIr-NS**, respectively.

RuIr-NC and **RuIr-NS** show quite similar XANES profiles below 1.40 V and distinct XANES profiles at 1.8 V.

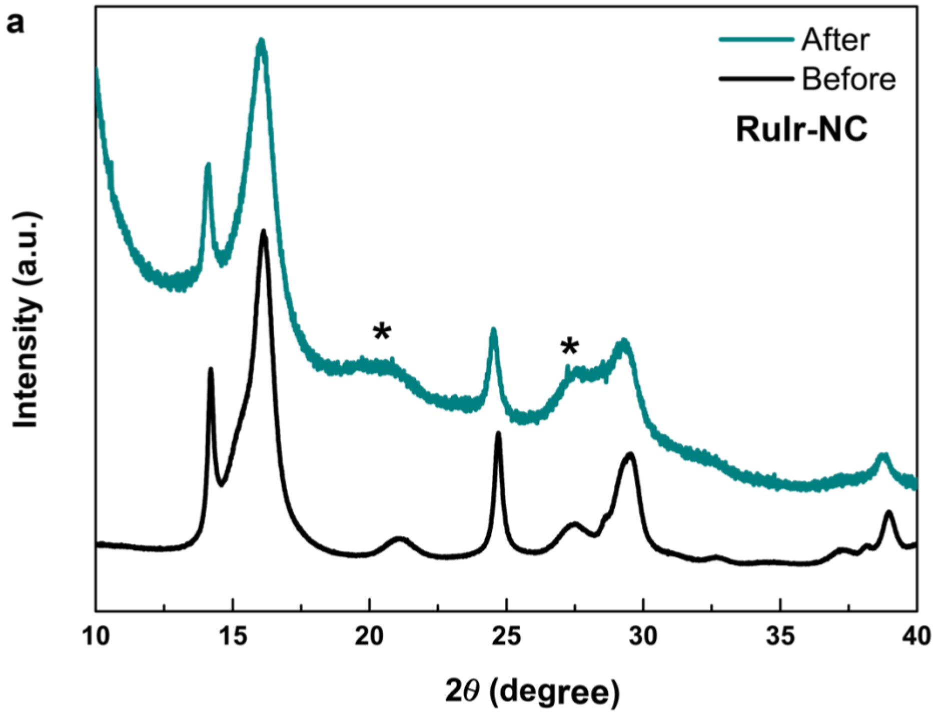

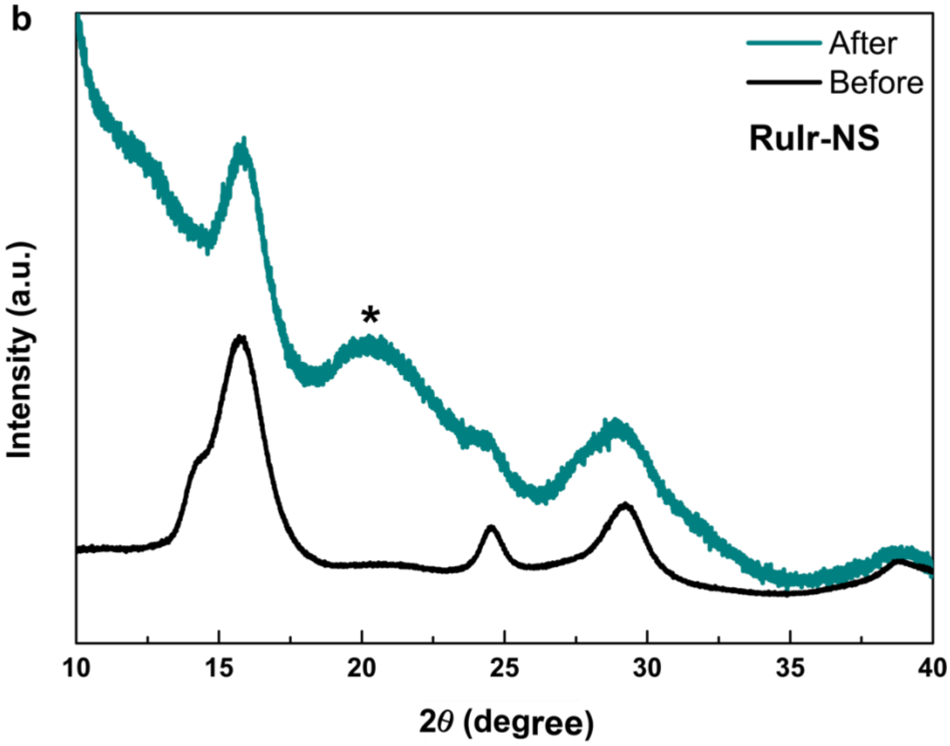

Fig. 30 Synchrotron XRD of RuIr catalysts after operando XANES. a, RuIr-NC. b, RuIr-NS. The signal marked with * comes from the sample capillary.

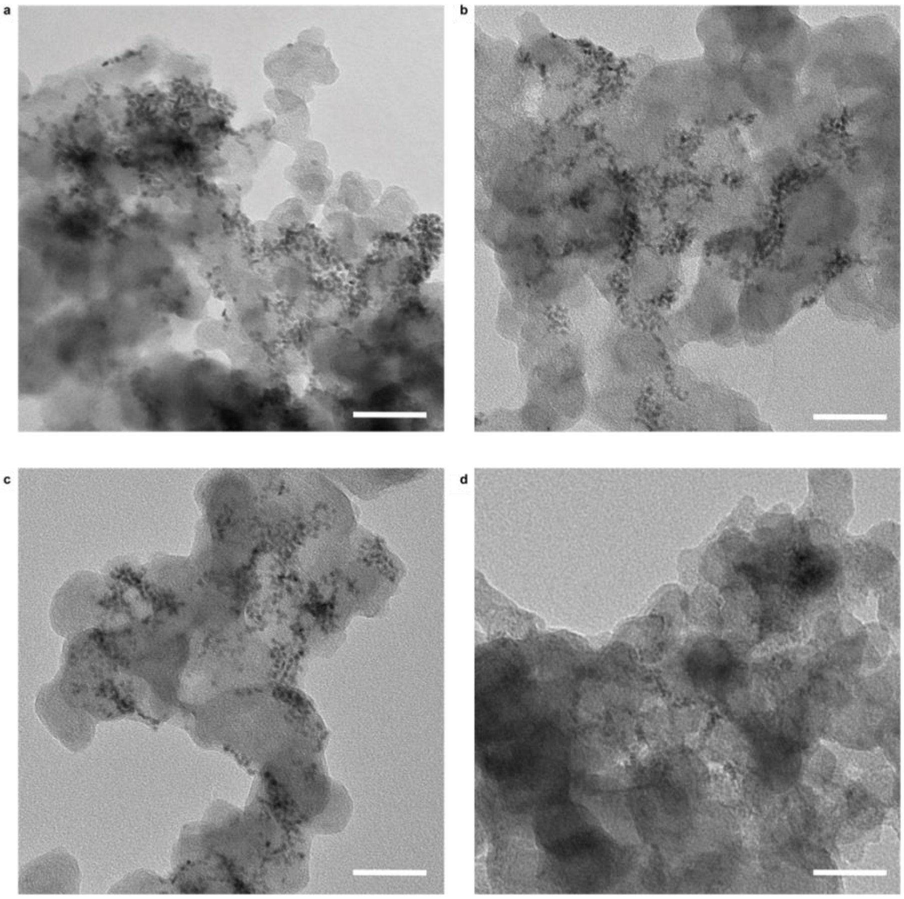

Fig. 31 Large-area TEM images of RuIr-NS after polarization scans to different potentials. a, Freshly prepared. **b,** At 1.25 V. **c,** At 1.40 V. **d,** At 1.80 V. The scale bar is 50 nm. At 1.80 V, the number and size of particles on the carbon support sharply decreased.

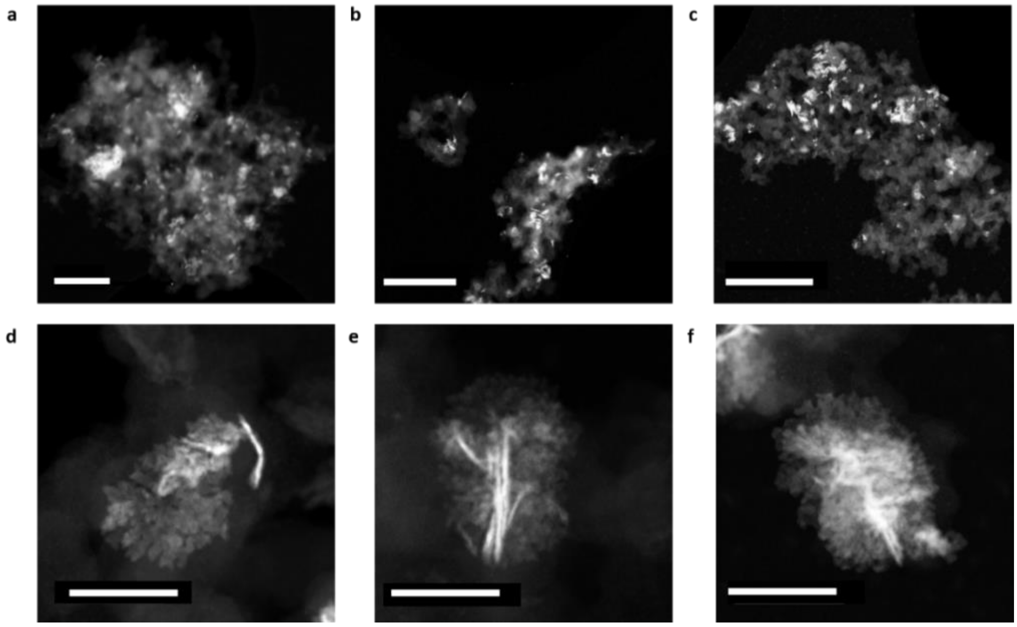

Fig. 32 STEM images of RuIr-NC after polarization scans to different potentials. a and **d,** 1.2 V. **b** and **e**, 1.40 V. **c** and **f**, 1.80 V. The scale bars for **a-c** and **d-f** are 500 and 50 nm, respectively. The size distributions at 1.2 V, 1.4 V and 1.8 V are 53 ± 9, 51± 15 and 54 ± 13 nm. The nanocoral maintained its morphology and size up to 1.80 V.

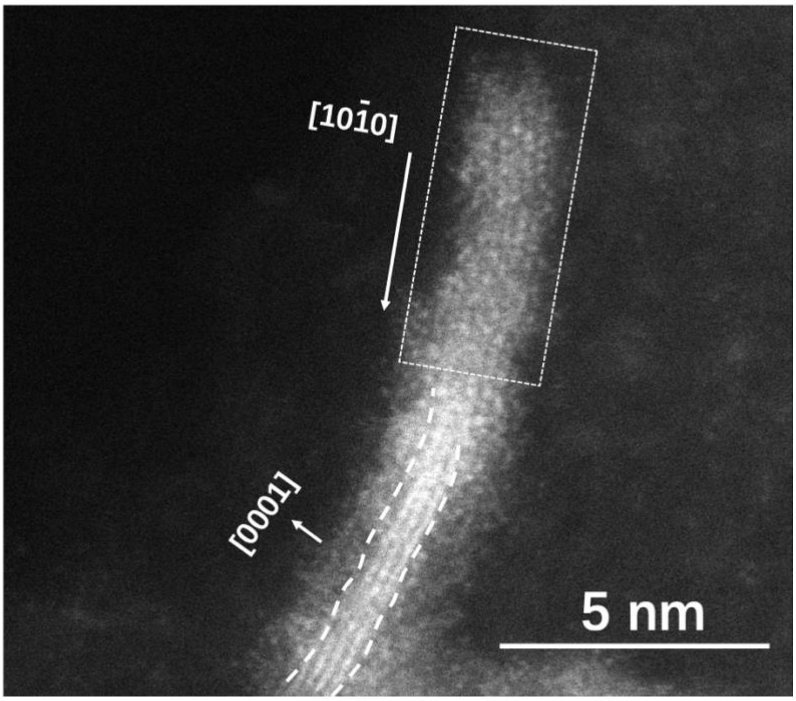

Fig. 33 HAADF-STEM image view from the [01$\bar{1}$0] direction of RuIr-NC at 1.8 V.

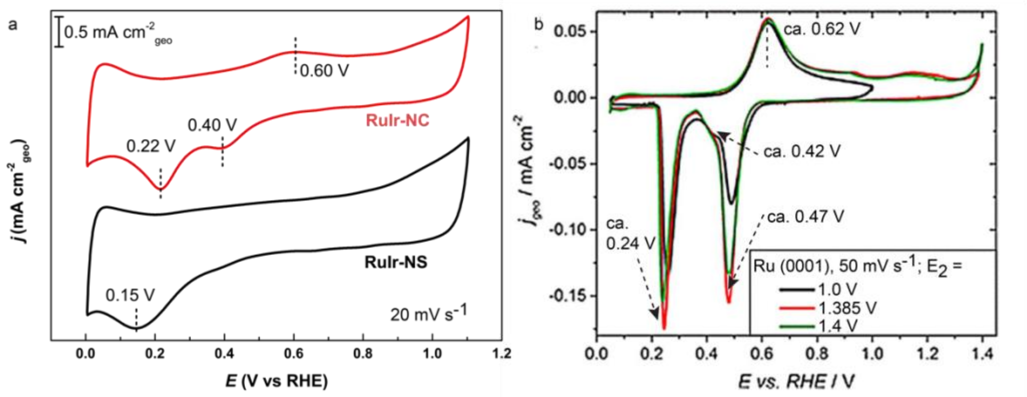

Fig. 34 CVs of RuIr catalysts and single-crystalline Ru(0001) electrodes in Ar-saturated 0.05 M H_2SO_4. a, CVs of **RuIr-NC** and **RuIr-NS** recorded at a scan rate of 20 mV/s. **b,** CVs of single-crystalline Ru(0001) electrode reported in Ref (*20*). Compared to **RuIr-NS**, the CV of **RuIr-NC** is more similar to that of single-crystalline Ru (0001) electrode, showing one oxidation peak at ca. 0.60 V, two reduction peaks at ca. 0.22 and 0.40V, respectively. Due to the nano effect and / or Ir doping, the peaks are more broaden than those of the single-crystalline electrode.

Table 1 Selected catalysts with high OER performance in an acidic electrolyte.

Catalysts	Electrolyte	Catalysts loading	substrate	Overpotential @ 10 mA cm^{-2}_{geo} (mV)	Durability test by CP method	Ref.
**$IrO_x/SrIrO_3$**	0.5 M H_2SO_4	5 × 5 mm^2 film	Cu wire attached	270–290	30 h @ 10 mA cm^{-2}	*(21)*
**6H-$SrIrO_3$**	0.5 M H_2SO_4	0.9 mg_{oxide} cm^{-2}	GC-RDE[1]	248	30 h @ 10 mA cm^{-2}	*(22)*
**$Pb_2Ir_2O_7$**	0.1 M $HClO_4$	0.2 mg_{oxide} cm^{-2}	Ti	>330	/	*(23)*
**$Y_2Ir_2O_7$**	0.1 M $HClO_4$	0.102 mg_{oxide} cm^{-2}	GC-RDE	/	/	*(24)*
**$BaYIrO_6$**	0.1 $HClO_4$	0.015–30 mg_{oxide} cm^{-2}	Au	315	1 h @ 1 mA cm^{-2}	*(25)*
**$W_{0.57}Ir_{0.43}O_{3-\delta}$**	1.0 M H_2SO_4	Ca. 0.77 mg_{oxides} cm^{-2}	FTO[2] GC-RDE	370	0.6 h @ 10 mA cm^{-2}	*(26)*
**IrO_x NPs/ATO [3]**	0.05 M H_2SO_4	0.010 mg_{Ir} cm^{-2}	ATO	ca.450	15 h @ 1 mA cm^{-2}	*(27)*
**$IrNiO_x$ core−shell particles**	0.05 M H_2SO_4	0.010 mg_{Ir} cm^{-2}	ATO	>330	20 h @ 1 mA cm^{-2}	*(28)*
**Ir nanowires**	0.5 M $HClO_4$	0.031 mg_{Ir} cm^{-2}	GC-RDE	270	~6.9 h @5 mA cm^{-2}	*(29)*
	0.1 M $HClO_4$			283	5.6 h @ 10 mA cm^{-2}	
**laminar Ir**	0.5 M H_2SO_4	0.0115 mg_{Ir} cm^{-2}	GC-RDE	270	8 h @ 1 mA cm^{-2}	*(30)*
**Ir NPs/3D graphite foam**	0.5 M H_2SO_4	0.82 mg_{Ir} cm^{-2}	CC[4]	290	30 h @ 10 mA cm^{-2}	*(31)*
**Ir−Fe NPs**	0.5 M $HClO_4$	0.092 mg_{Ir} cm^{-2}	GC-RDE	278	3.8 h @10 mA cm^{-2}	*(32)*
**Ir-SA@Fe@NCNT**	0.5 M H_2SO_4	0.00114 mg_{Ir} cm^{-2}	GC-RDE	250	12 h @ 10 mA cm^{-2}	*(33)*

**$Y_{1.8}Cu_{0.2}Ru_2O_{7-\delta}$**	1.0 N H_2SO_4	0.0125 mg_{oxide} cm^{-2}	GC-RDE	< 380	6 h @ 1.0 mA cm^{-2}	*(34)*
**Co-doped RuO_2**	0.5 M H_2SO_4	/	GC-RDE	169	50 h @ 1.0 mA cm^{-2}	*(35)*
**Li-$IrSe_2$**	0.5 M H_2SO_4	/	CP	180	10 h @ 10 mA cm^{-2}	*(36)*
**Np-IrO_2**	0.5 M H_2SO_4	1.0 mg cm^{-2}	Self-suppoted	240	30 h @ 100 mA cm^{-2}	*(37)*
**AuIr@CNT**	0.5 M H_2SO_4	/		257	12 h @ 10 mA cm^{-2}	*(38)*
**IrNi NCs** [7]	0.1 $HClO_4$	0.0125 mg_{Ir} cm^{-2}	CP[5]	280	2 h @ 5 mA cm^{-2}	*(39)*
**IrCoNi PHNCs**	0.1 M $HClO_4$	0.01 mg_{Ir} cm^{-2}	GC-RDE	303	3.3 h @ 5 mA cm^{-2}	*(40)*
**Co-RuIr**	0.1 M HClO4	/	GC-RDE	235	25 h @ 10 mA cm^{-2}	*(41)*
**SrTi(Ir)O_3**	0.1 M $HClO_4$	0.28 mg_{oxide} cm^{-2}	GC-RDE	261	20 h @ 10 mA cm^{-2}	*(42)*
**Ru_xIr_{1-x} bulk alloy**	0.1 M $HClO_4$	Thin film	Ti	>223	/	*(27)*
**Rutile-IrO_2 NPs**	0.1 M $HClO_4$	0.05 mg_{oxide} cm^{-2}	GC-RDE	>430	/	*(9)*
**Ru−Au 3-D branches**	0.1 M $HClO_4$	0.408 mg_{metal} cm^{-2}	GC-RDE	220	/	*(43)*
**RuO_2 nanowires/g-CN_x**	0.5 M H_2SO_4	0.062 mg_{oxide} cm^{-2}	GC-RDE	250	/	*(10)*
**Cu−doped RuO_2 hollow porous polyhedral**	0.5 M H_2SO_4	0.275 mg_{oxide} cm^{-2}	GC-RDE	188	8 h @ 10 mA cm^{-2}	*(12)*
**$CaCu_3Ru_4O_{12}$**	0.5 M H_2SO_4	0.25 mg_{oxide} cm^{-2}	GC-RDE	171	24h @ 10 mA cm^{-2}	*(44)*
**Na-doped $SrRuO_3$**	0.1 M $HClO_4$	0.5 mg_{oxide} cm^{-2}	GC-RDE	~170	/	*(45)*
**$Cr_{0.4}Ru_{0.6}O_2$**	0.5 M H_2SO_4	0.279 mg_{oxide} cm^{-2}	GC-RDE	178	10 h @ 10 mA cm^{-2}	*(46)*
**Rutile-RuO_2 NPs**	0.1 M $HClO_4$	0.05 mg_{oxide} cm^{-2}	GC-RDE	>420	/	*(9)*
**Ba[Co-polyoxometalates**	1 M H_2SO_4	11–16 mg filled in 0.07 cm^2 RDE	RDE	361	/	*(47)*

**]/carbon paste blend**						
**N−WC**	0.5 M H_2SO_4	10 mg cm^{-2}	CP	ca.260	1 h @ 10 mA cm^{-2}	*(13)*
**Co_3O_4**	0.5 M H_2SO_4	12.5 mg_{oxide} cm^{-2}	CP	370	12 h @ 10 mA cm^{-2}	*(48)*
**ONPPGC/OCC**	0.5 M H_2SO_4	/	CC	470	/	*(49)*
**NC−CNT/CoP**	0.5 M H2SO4	/	CC	350	/	*(50)*
**Co doped-MoS_2**	0.5 M H_2SO_4	/	/	540	/	*(51)*
**$Ru_{1-x}Zn_xO_2$ NPs**	0.10 M $HClO_4$/0.15 M NaCl	1–2 mg mg_{oxide} cm^{-2}	Ti	>330	/	*(14)*
**Ru-Te nanorod**	0.5 M H_2SO_4	0.102 mg_{metal} cm^{-2}	GC-RDE	245	/	*(52)*
**Ru_1-Pt_3Cu**	0.1 M HClO4	0.0163 mg_{Pt+Ru} cm^{-2}	GC-RDE	220	28 h @ 10 mA cm^{-2}	*(53)*
**$Y_2Ru_2O_{7-\delta}$**	0.1 M $HClO_4$	~ 0.026 mg_{oxide} cm^{-2}	GC-RDE	>370	8 h @ 1 mA cm^{-2}oxides	*(54)*
**RuIr-NC**	0.05 M H_2SO_4	0.05 mg_{metal} cm^{-2}	GC-RDE	170	122 h @ 1 mA cm^{-2}	This paper
					40 h @ 10 mA cm^{-2}	

[1] GC: glassy carbon

[2] FTO: Fluorine doped tin oxide

[3] ATO: antimony-doped tin oxide

[4] Glassy carbon electrode

[5] CC: carbon cloth

[6] CP: carbon paper

[7] NCs: nanocrystal

Table 2 First shell fitting of EXAFS of Ir *L*3-edge of RuIr-NC and RuIr-NS.

**Sample**	**Shell**	***N***	**σ^2/Å^2**	**ΔE_0/eV**	***R*/Å**	***R*-factor**
**Ir bulk**	Ir−Ir	12[1]	0.003	9.28	2.71(1)	0.003
**RuIr-NC[2]**	Ir−Ru	8.9 ± 1.0 [3]	0.005(6)[4]	8.84 ± 0.97	2.68(4)	0.011
**RuIr-NS**	Ir−Ru	8.9 ± 1.3	0.006 (8)	8.91 ± 1.22	2.68(6)	0.015

[1] coordination (N) is fixed at 12 for Ir bulk.

[2] Debye−Waller parameters (σ^2), N, distance (*R*) and energy shift (ΔE_0) were kept unrestricted for RuIr catalysts.

[3][4] Mean ± standard deviation

Table 3 Amount of Ru species in RuIr catalysts at different potentials derived from an LCF of normalized XANES spectra

Potential (V vs RHE)	RuIr-NC				RuIr-NS			
	components		Goodness-of-fit		components		Goodness-of-fit	
	Ru	RuO_2	*R*-factor [1]	χ^2	Ru	RuO_2	*R*-factor	χ^2
**OCP**	0.898(5)[2]	0.102(4)	0.00057	0.021	0.816(7)	0.184(6)	0.0013	0.049
**1.25**	0.776(5)	0.224(3)	0.00042	0.016	0.787(7)	0.213(6)	0.0013	0.049
**1.30**	0.757(5)	0.243(4)	0.00053	0.020	0.770(7)	0.230(6)	0.0011	0.044
**1.35**	0.732(5)	0.268(3)	0.00042	0.016	0.747(7)	0.253(5)	0.0011	0.041
**1.40**	0.704(5)	0.296(3)	0.00041	0.015	0.740(7)	0.260(5)	0.00093	0.035
**1.45**	0.688(5)	0.312(3)	0.00042	0.015	0.677(6)	0.323(5)	0.00086	0.034
**1.50**	0.661(4)	0.339(2)	0.00024	0.0094	0.666(6)	0.334(4)	0.00066	0.025
**1.55**	0.648(4)	0.352(3)	0.00029	0.011	0.558(6)	0.442(4)	0.00064	0.026
**1.60**	0.635(4)	0.365(3)	0.00028	0.011	0.463(5)	0.537(3)	0.00037	0.015
**1.65**	0.621(4)	0.379(2)	0.00020	0.0080	0.350(5)	0.650(3)	0.00033	0.014
**1.70**	0.618(4)	0.382(3)	0.00025	0.0097	0.302(5)	0.698(3)	0.00038	0.017
**1.75**	0.607(4)	0.393(2)	0.00019	0.0075	0.277(6)	0.723(4)	0.00043	0.019
**1.80**	0.600(4)	0.400(2)	0.00018	0.0069	0.258(6)	0.742(4)	0.00046	0.021

[1] Residual factor based on $\sum [(\text{data} - \text{fit})^2] / \sum (\text{data}^2)$

[2] Mean ± standard deviation

Table 4 The atomic percentage (%) of Ru and Ir in the as-prepared RuIr catalysts and RuIr catalysts during OER.

		**EDX @ JEOL ARM-200 kV**			**XRF**	**XPS**
	As-prepared	1.2 V	1.4 V	1.8 V	As-prepared	As-prepared
**RuIr-NS**	95.9 / 4.1	94.5 / 5.4	95.2 / 4.8	83.8 / 16.2	93.5 / 6.5	92.0 / 8.0
**RuIr-NC**	96.0 / 4.0	96.0 / 4.0	93.8 / 6.2	92.8 / 7.8	93.6 / 6.4	93.5 / 6.5

[1] Lab XPS uses Mg *K*α source energy with a probe depth of approximately 1 nm. This reveals the surface composition is similar to the bulk composition.

Table 5. Comparison of the electrolyzer built with bifunctional electrocatalysts in acidic media. [1]

Catalysts	electrolyte	Catalysts loading	substrate	Overall potential @geometric current density	Durability	Ref
Ir nanowire	0.1 M $HClO_4$	0.03 mg cm^{-2}	CC	1.62 V @ 10 mA cm^{-2}	11 h @ 10 mA cm^{-2}	*(29)*
Ir NPs				1.73 V @ 10 mA cm^{-2}	/	
Co-MoS_2	0.5 M H_2SO_4	/	CP	1.90 V @ 10 mA cm^{-2}	/	*(51)*
NC-CNT/CoP	0.5 M H_2SO_4	/	CC	1.66 V @ 10 mA cm^{-2}	/	*(50)*
IrCoNi PHNCs	0.5 M H_2SO_4	/	CP	1.60 V @ 4.66 mA cm^{-2}	/	*(40)*
IrNi NCs[2]	0.1 M $HClO_4$	0.0125 mg_{Ir} cm^{-2}	CP	1.58 V @ 10 mA cm^{-2}	/	*(39)*
**Ir-SA@Fe@N CNT**	0.5 M H_2SO_4	0.00114 mg_{Ir} cm^{-2}		1.51 V @ 10 mA cm^{-2}	12 h@ 10 mA cm^{-2}	*(33)*
**AuIr@CNT**	0.5 M H_2SO_4	/		1.51 V @ 10 mA cm^{-2}	50 h @ 10 mA cm^{-2}	*(38)*
**Li-IrSe$_2$**	0.5 M H_2SO_4	/		1.51 V @ 10 mA cm^{-2}	24 h @ 10 mA cm^{-2}	*(36)*
**np-IrO$_2$**	0.5 M H_2SO_4	1.0 mg cm^{-2}		1.52 V @ 10 mA cm^{-2}	40 h @ 100 mA cm^{-2}	*(37)*
ONPPGC/GC	0.5 M H_2SO_4	/	CC	1.75@ 10 mA cm^{-2}	10 h @ 5 mA cm^{-2}	*(49)*
Ru-Te nanorods	0.5 M H_2SO_4	/	CP	1.52@ 10 mA cm^{-2}	24 h @ 5 mA cm^{-2}	*(52)*
Co-RuIr	0.1 M $HClO_4$	/	CP	1.52@ 10 mA cm^{-2}	25 h @ 10 mA cm^{-2}	*(41)*
**RuIr-NC**	0.05 M H_2SO_4	0.15 mg cm^{-2}	CP	1.485@ 10 mA cm^{-2}	120 h @ 10 mA cm^{-2}	This paper

[1] All the LSV are *i*R-corrected in these references.

[2] NCs: nanocrystals

Supplementary Movie S1. 3-D tomography construction of an optical **RuIr-NC** particle.

Supplementary Movie S2. Overall water splitting on a two-electrode configuration using **RuIr-NC** for both OER and HER and commercial benchmark couple (Pt/C for HER and highly conductive IrO_2 nanocatalysts for OER in 0.05 M H_2SO_4.

Supplementary References

1. K. J. Batenburg, J. S. in *IEEE International Conference on Image Processing.* 133-136.

2. Batenburg, B. et al. 3D imaging of nano1materials by discrete tomography. *Ultramicroscopy* **109**, 730-740 (2009).

3. van Aarle, W. et al. The ASTRA Toolbox: A platform for advanced algorithm development in electron tomography. *Ultramicroscopy* **157**, 35-47 (2015).

4. De Backer, A. et al. StatSTEM: An efficient approach for accurate and precise model-based quantification of atomic resolution electron microscopy images. *Ultramicroscopy* **171**, 104-116 (2016).

5. Kirkland, E. Advanced computing in electron microscopy. *J. Appl. Cryst.* **32**, 378-379 (1999).

6. Ravel, B. and Newville, M. ATHENA, ARTEMIS, HEPHAESTUS: data analysis for X-ray absorption spectroscopy using IFEFFIT. *J. Synchro. Radiat.* **12**, 537-541 (2005).

7. Ressler, T., Wong, J., Roos, J. and Smith, I. L. Quantitative speciation of Mn-bearing particulates emitted from autos burning (methylcyclopentadienyl)manganese tricarbonyl-added gasolines using XANES spectroscopy. *Environ. Sci. Technol.* **34**, 950-958 (2000).

8. Sugawara, Y., Yadav, A. P., Nishikata, A. and Tsuru, T. EQCM study on dissolution of ruthenium in sulfuric acid. *J. Electrochem. Soc.* **155**, B897-B902 (2008).

9. Lee, Y., Suntivich, J., May, K., Perry, E. and Yang, S. Synthesis and Activities of Rutile IrO2 and RuO2 Nanoparticles for Oxygen Evolution in Acid and Alkaline Solutions. *J. Phys. Chem. Lett.* **3**, 399-404 (2012)

10. T. Bhowmik, M. K. Kundu, S. Barman, Growth of one dimensional RuO_2 nanowires on g-carbon nitride: An active and stable bifunctional electrocatalyst for hydrogen and oxygen evolution reactions at all pH values. *ACS. Appl. Mater. Interfaces* **57**, 28678–28688 (2016).

11. Rao, R. R. et al. Towards identifying the active sites on RuO2(110) in catalyzing oxygen evolution. *Energy Environ. Sci.* **10**, 2626-2637 (2017).

12. J. Su *et al.*, Assembling ultrasmall copper-doped ruthenium oxide nanocrystals into hollow porous polyhedra: Highly robust electrocatalysts for oxygen evolution in acidic media. *Adv. Mater.* **30**, 1801351-1801358 (2018).

13. N. Han *et al.*, Nitrogen-doped tungsten carbide nanoarray as an efficient bifunctional electrocatalyst for water splitting in acid. *Nat. Com.* **9**, 924 (2018).

14. V. Petrykin, K. Macounova, O. A. Shlyakhtin, P. Krtil, Tailoring the selectivity for electrocatalytic oxygen evolution on ruthenium oxides by zinc substitution. *Angew. Chem. Int. Ed. Engl.* **49**, 4813-4815 (2010)

15. R. Frydendal, E. A. Paoli, I. Chorkendorff, J. Rossmeisl, I. E. L. Stephens, Toward an Active and Stable Catalyst for Oxygen Evolution in Acidic Media: Ti-Stabilized MnO_2. *Adv. Energy Mater.* **5**, 1500991 (2015).

16. Wei, C. et al. Recommended Practices and Benchmark Activity for Hydrogen and Oxygen Electrocatalysis in Water Splitting and Fuel Cells. *Adv. Mater*. e1806296 (2019).

17. Minguzzi, A., Lugaresi, O., Achilli, E., Locatelli, C., Vertova, A., Ghigna, P. and Rondinini, S. *Chem. Sci.*, 5, 3591-3597 (**2014**)

18. Nong, H. N. et al. A unique oxygen ligand environment facilitates water oxidation in hole-doped IrNiOx core-shell electrocatalysts. *Nat. Catal.* **1**, 841-851 (2018).

19. Choy, J., Kim, D., Hwang, S., Demazeau, G and Jung, D. *J. Am. Chem. Soc.*, 117, 8557-8566 (**1995**).

20. Özer E, Spöri C, Reier T and Strasser P. Iridium (111), iridium (110), and ruthenium (0001) single crystals as model catalysts for the oxygen evolution reaction: insights into the electrochemical oxide formation and electrocatalytic activity. *ChemCatChem*, **9**, 597-603 (2017).

21. Seitz, L. C. et al. A highly active and stable IrOx/SrIrO3 catalyst for the oxygen evolution reaction. *Science* **353**, 1011-1014 (2016).

22. Yang, L. et al. Efficient oxygen evolution electrocatalysis in acid by a perovskite with face-sharing IrO6 octahedral dimers. *Nat. Commun.* **9**, 5236 (2018).

23. W. Sun *et al.*, OER activity manipulated by IrO_6 coordination geometry: an insight from pyrochlore iridates. *Sci. Rep.* **6:38429**, 1-10 (2016).

24. D. Lebedev *et al.*, Highly active and stable iridium pyrochlores for oxygen evolution reaction. *Chem. Mater.* **29**, 5182-5191 (2017).

25. O. Diaz-Morales *et al.*, Iridium-based double perovskites for efficient water oxidation in acid media. *Nat. Commun.* **7**, 12363 (2016).

26. S. Kumari *et al.*, A low-noble-metal W1-x$Ir_xO_{3-\delta}$ water oxidation electrocatalyst for acidic media via rapid plasma synthesis. *Energy Environ. Sci.* **10**, 2432-2440 (2017).

27. Oh, H.-S. et al. Electrochemical Catalyst–Support Effects and Their Stabilizing Role for IrO x Nanoparticle Catalysts during the Oxygen Evolution Reaction. *J. Am. Chem. Soc.* **138**, 12552-12563 (2016).

28. H. N. Nong *et al.*, Oxide-supported IrNiO(x) core-shell particles as efficient, cost-effective, and stable catalysts for electrochemical water splitting. *Angew. Chem. Int. Ed. Engl.* **54**, 2975-2979 (2015).

29. L. Fu, F. Yang, G. Cheng, W. Luo, Ultrathin Ir nanowires as high-performance electrocatalysts for efficient water splitting in acidic media. *Nanoscale* **10**, 1892-1897 (2018).

30. Y. Pi, N. Zhang, S. Guo, J. Guo, X. Huang, Ultrathin laminar Ir superstructure as highly efficient oxygen evolution electrocatalyst in broad pH range. *Nano Lett.* **16**, 4424-4430 (2016).

31. J. Zhang *et al.*, Iridium nanoparticles anchored on 3D graphite foam as a bifunctional electrocatalyst for excellent overall water splitting in acidic solution. *Nano Energy* **40**, 27-33 (2017).

32. L. Fu, P. Cai, G. Cheng, W. Luo, Colloidal synthesis of iridium-iron nanoparticles for electrocatalytic oxygen evolution. *Sustain. Energy Fuels* **1**, 1199-1203 (2017).

33. Luo, F., Hu, H., Zhao, X., Yang Z., Zhang, Q., Xu, J., Kaneko, T., Yoshida, Y., Zhu, C., Cai, W., *Nano Lett.* **20**, 2120–2128 (2020).

34. Kuznetsov, D., Naeem, M., Kumar, P., Abdala, P., Fedorov, A. and Müller, C. *J. Am. Chem. Soc.*, **142**, 7883-7888 (2020).

35. Tian, Y., Wang, S., Velasco, E., Yang, Y., Cao, L., Zhang, L., Li, X., Lin, Y., Zhang, Q. and Chen, L. *iScience*, **23**, 100756 (2020).

36. Zheng, T., Shang, C., He, Z., Wang, X., Cao, C., Li, H., Si, R., Pan, B., Zhou, S. and Zeng, J. *Angew. Chem. Int. Ed.* **7**, 14764-14769 (2019).

37. Hegde, G., Ghosh, A., Badam, R., Matsumi, N. and Sundara, R. *ACS Appl. Energy Mater.* 3, 3736-3744 (2020).

38. S. Hao, Y. Wang, G. Zheng, L. Qiu, N. Xu, Y. He, L. Lei, X. Zhang, *Appl. Catal. B* **266**， 118643 (2020).

39. Q. S. Yecan Pi, Pengtang Wang, Jun Guo, Xiaoqing Huang, General formation of monodisperse IrM (M = Ni, Co, Fe) bimetallic nanoclusters as bifunctional electrocatalysts for acidic overall water splitting. *Adv. Func. Mater* **27**, 8 (2017).

40. J. Feng *et al.*, Iridium-based multimetallic porous hollow nanocrystals for efficient overall-water-splitting catalysis. *Adv. Mater.* **29**, (2017).

41. Shan, J., Ling, T., Davey, K., Zheng, Y., Qiao, S. Transition-metal-doped RuIr bifunctional nanocrystalsfor overall water splitting in acidic environments. *Adv. Mater.* **31**, 1900510 (2019).

42. Chen, H., Shi, L., Liang, X., Wang, L., Asefa, T., Zou, X. Optimization of Active Sites via Crystal Phase, Composition, and Morphology for Efficient Low-Iridium Oxygen Evolution Catalysts. *Angew. Chem. Int. Ed.* **132**, 1-6 (2020).

43. Gloag, L. et al. Three-Dimensional Branched and Faceted Gold-Ruthenium Nanoparticles: Using Nanostructure to Improve Stability in Oxygen Evolution Electrocatalysis. *Angew. Chem. Int. Ed. Engl.* **57**, 10241-10245 (2018).

44. X. Miao, L. Zhang, L. Wu, Z. Hu, L. Shi, Zhou, S. Quadruple perovskite ruthenate as a highly efficient catalyst for acidic water oxidation. *Nat. Commun.* 10, 1, (2019).

45. M. Retuerto, L. Pascual, F. Calle-Vallejo, P. Ferrer, D. Gianolio, A. G. Pereira, J. Torrero, M. Fernández-Díaz, P. Bencok, M. Peña, J. Fierro, S. Rojas. Na-doped ruthenium perovskite electrocatalysts with improved oxygen evolution activity and durability in acidic media. *Nat. Commun.* **10**, 2041 (2019).

46. Lin, Y. et al. Chromium-ruthenium oxide solid solution electrocatalyst for highly efficient oxygen evolution reaction in acidic media. *Nat. Commun.* **10**, 162 (2019).

47. Blasco-Ahicart, M., Soriano-López, J., Carbó, J., Poblet, J. and Galan-Mascaros, J. Polyoxometalate electrocatalysts based on earth-abundant metals for efficient water oxidation in acidic media. *Nat. Chem.* **10**, 24-30 (2018).

48. X. L. Yang *et al.*, Highly acid-durable carbon coated Co_3O_4 nanoarrays as efficient oxygen evolution electrocatalysts. *Nano Energy* **25**, 42-50 (2016).

49. S. L. Jianping Lai, Fengxia Wu, Muhammad Saqib, Rafael Luque, Guobao Xu, Unprecedented metal-free 3D porous carbonaceous electrodes for full water splitting. *Energy Environ. Sci.* **9**, 5 (2016).

50. C. Guan *et al.*, Metal-organic framework-derived integrated nanoarrays for overall water splitting. *J. Mater. Chem. A* **6**, 9009-9018 (2018).

51. Q. Xiong *et al.*, One-step synthesis of cobalt-doped MoS_2 nanosheets as bifunctional electrocatalysts for overall water splitting under both acidic and alkaline conditions. *Chem. Com.* **54**, 3859-3862 (2018).

52. Wang, J., Han, L., Huang, B., Shao, Q., Xin, H., Huang, X. Amorphization activated ruthenium-tellurium nanorods for efficient water splitting. *Nat. Commun.* 10, 5692 (2019).

53. Yao, Y., Hu, S., Chen, W., Huang, Z., Wei, W., Yao, T., Liu, R., Zang, X., Wu, G., Yuan, W., Zhu, B., Liu, W., Li, Z., He, D., Xue, Z., Wang, Y., Zheng, X., Dong, J., Chang, C., Chen, Y., Hong, X., Luo, J., Wei, S., Li, W., Strasser, P., Wu, Y. and Li, Y. Engineering the electronic structure of single atom Ru sites via compressice strain boosts acidic water oxidation electrocatalysis. *Nat. Catal.*, **2**, 304-313 (2019).

54. Kim, J. et al. High-Performance Pyrochlore-Type Yttrium Ruthenate Electrocatalyst for Oxygen Evolution Reaction in Acidic Media. *J. Am. Chem. Soc.* **139**, 12076-12083 (2017).

55. A. Grimaud, A. Demortière, M. Saubanère, W. Dachraoui, M. Duchamp, M.-L. Doublet, J.-M. Tarascon, Activation of surface oxygen sites on an iridium-based model catalyst for the oxygen evolution reaction. *Nat. Energy*, **2**, 16189, (2017).

56. O. Diaz-Morales, S. Raaijman, R. Kortlever, P. J. Kooyman, T. Wezendonk, J. Gascon, W. T. Fu, M. T. M. Koper, Iridium-based double perovskites for efficient water oxidation in acid media. *Nat. Commun.* **7**, 12363, (2016).
