# Supplementary material for: Efficient overall water splitting in acid with anisotropic metal nanosheets
Source: Nat Commun. 2021 Feb 16;12:1145. doi: 10.1038/s41467-021-20956-4 (PMC7887272; doi:10.1038/s41467-021-20956-4)
Supplement: Supplementary file 5 — Description of additional supplementary files [file 41467_2021_20956_MOESM5_ESM.docx]

Description of additional supplementary information:

Title: Supplementary Movie S1.

Description: 3-D tomography construction of an optical RuIr-NC particle.

Title: Supplementary Movie S2.

Description: Overall water splitting on a two-electrode configuration using RuIr-NC for both OER and HER and commercial benchmark couple (Pt/C for HER and highly conductive IrO2 nanocatalysts for OER in 0.05 M H2SO4
